# Early Nutrition, Blood Amino Acids and Outcomes in Preterm Babies: Secondary Cohort Analysis of the ProVIDe RCT

**DOI:** 10.3390/nu18101517

**Published:** 2026-05-09

**Authors:** Barbara Cormack, Amelia van Duinen, Nadia Ford, Yannan Jiang, Mark de Hora, Natasha Heather, Frank Bloomfield

**Affiliations:** 1 Liggins Institute, University of Auckland, Auckland 1023, New Zealand; 2 Starship Child Health, Auckland City Hospital, Auckland 1023, New Zealand; 3 Department of Statistics, University of Auckland, Auckland 1010, New Zealand; 4 LabPlus, Auckland 1140, New Zealand; mdehora@adhb.govt.nz (M.d.H.); nheather@adhb.govt.nz (N.H.)

**Keywords:** parenteral nutrition, neurodevelopment, preterm, amino acids, lipid

## Abstract

Background: Providing optimal parenteral nutrition to extremely preterm babies in the first week after birth is challenging, and different strategies may be associated with both short- and long-term outcomes. Methods: In a secondary cohort analysis of the ProVIDe trial, a multicentre, randomised, controlled trial in extremely-low-birthweight babies of increased amino acid intake in the first five days after birth, we explored the associations between parenteral amino acid and lipid intakes and blood spot amino acid concentrations, clinical outcomes and neurodevelopment. The cohort comprised 382 babies born in six New Zealand hospitals of whom 342 survived to 28 days. Nutritional intake data in the first week and newborn metabolic screening data on days 1, 5, 14, and 28 were retrieved, and 294 children were assessed for neurodevelopmental outcome at 2 years’ corrected age. Results: Blood spot amino acid concentrations were positively associated with amino acid intake (*p* < 0.005). Higher amino acid intakes were associated with increased odds (OR), 95% confidence intervals (CIs) of bronchopulmonary dysplasia (tyrosine: OR 2.2, CI 1.2–3.9; proline: OR 2.3, CI 1.3–4.0), patent ductus arteriosus and probable sepsis. No significant associations were found for necrotising enterocolitis. Higher lipid intakes were associated with lower odds of intraventricular haemorrhage (0.33 [0.16, 0.66]), bronchopulmonary dysplasia (0.31 [0.13, 0.73]) and retinopathy of prematurity (0.29 [0.12, 0.72]). Unlike short-term outcomes, neurodevelopment did not differ according to blood spot or intake quartile for any amino acid in week 1. Conclusions: Parenteral nutritional intakes in the first week after birth are associated with short-term outcomes. Further research is needed to optimise the composition of amino acid solutions. Trial Registration: ACTRN12612001084875, (accessed on 10 October 2012).

## 1. Introduction

Extremely preterm babies face significantly higher risks of morbidity, mortality and neurodisability compared to those born at term [1,2,3]. Nutrition, a modifiable factor, is also closely linked to these outcomes [4]. For extremely-low-birthweight babies (ELBW; <1000 g), parenteral nutrition is essential due to limited nutrient reserves, high metabolic demand and gut immaturity. Although used since the 1960s, preterm parenteral nutrition solutions are restricted to only a few available preparations, formulated either based on amino acid profile of cord blood or to reflect the amino acid composition of human milk and have not been substantially updated for many years [5,6]. The optimal macronutrient composition remains unclear, leading to wide variations in practice [7]. Despite consensus recommendations, target intakes often are unmet, contributing to faltering growth and suboptimal clinical and neurodevelopmental outcomes [8,9,10]. Normative plasma amino acid concentrations for ELBW babies in the first four weeks are unknown but one small trial indicates that early parenteral amino acid intakes are associated with plasma amino acid concentrations [11]. Some studies suggest that high amino acid intakes and blood amino acid concentrations may be associated with less optimal clinical and developmental outcomes [11,12,13].

Our aims were to determine blood spot amino acid concentrations in the first four weeks after birth in a cohort of ELBW babies enrolled in a randomised controlled trial and whether:

1. Parenteral amino acid intakes in the first week after birth are associated with blood spot amino acid concentrations.
2. Parenteral amino acid and fatty acid intakes in the first week after birth are associated with clinical outcome or neurodevelopment at 2 years’ corrected age (CA).
3. Blood spot amino acid concentrations in the first week are associated with clinical outcomes or neurodevelopment at 2 years’ CA.
4. Amino acid or lipid brand is associated with clinical outcomes or neurodevelopmental outcomes at 2 years’ CA.

## 2. Materials and Methods

**Figure 1:**
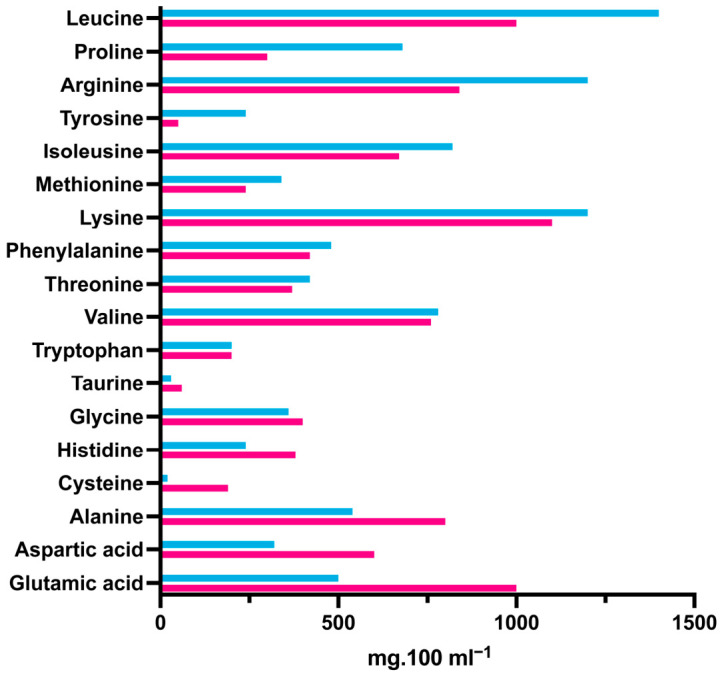
Comparison of the amino acid composition of Primene (pink bars) and TrophAmine (blue bars) parenteral amino acid solutions.

**Figure 2:**
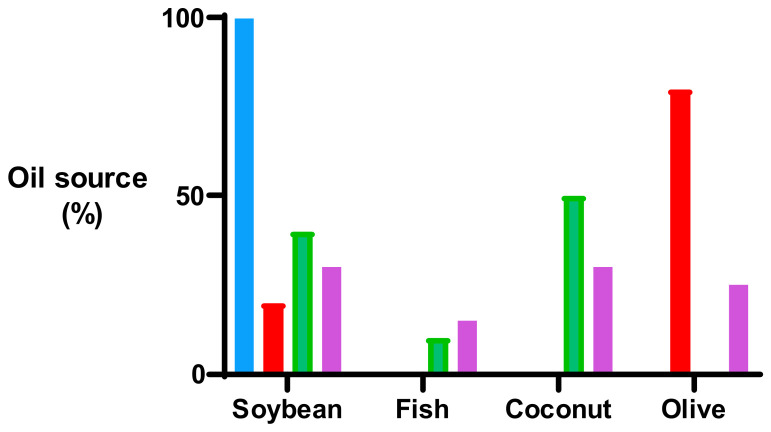
Oil source of four lipid emulsions. Blue bars represent Intralipid, red bars represent Clinoleic, green bars represent Lipoplus, and purple bars represent SMOFlipid.

This is a secondary cohort analysis of a subgroup of the 434 ELBW babies enrolled in the ProVIDe trial, a multicentre, triple-blind, placebo-controlled randomised trial (Australian New Zealand Clinical Trials Registry: ACTRN12612001084875 https://www.anzctr.org.au/Trial/Registration/TrialReview.aspx?id=363124&isReview=true (accessed on 1 March 2026); protocol and primary outcome published elsewhere [14,15]). In brief, babies were randomised (April 2014 to October 2018) within 24 h of birth to receive the intervention or placebo in addition to standard parenteral nutrition prescribed as per the local protocol. Both the intervention (an additional 1 g per day of protein as an amino acid solution (TrophAmine^®^, B. Braun New Zealand Pty Ltd., Auckland, New Zealand) and the placebo (0.45% saline) were delivered via an umbilical arterial catheter (UAC) for 120 h or until the UAC was removed. All babies also received parenteral nutrition including an amino acid solution, which was either TrophAmine^®^, B. Braun New Zealand Pty Ltd., Auckland, New Zealand or Primene^®^ Baxter Healthcare Limited, 33 Vestey Drive, Mount Wellington, Auckland, New Zealand, and lipid emulsion according to unit protocols. Intralipid is a 100% soybean oil-based lipid emulsion commonly used worldwide for many years. Newer lipid emulsions such as ClinOleic Baxter Healthcare Limited, 33 Vestey Drive, Mount Wellington, Auckland 1060, New Zealand and SMOFlipid Fresenius Kabi New Zealand Limited, Level 14, 188 Quay Street, Auckland 1010, New Zealand, also contain varying concentrations of soybean oil, olive oil, medium-chain triglycerides, and fish oil. The composition of the two amino acid solutions and the four lipid emulsions available for use in New Zealand at the time of the trial are shown in Figure 1 and Figure 2.

Inclusion criteria were birthweight <1000 g and the placement of a UAC. The ProVIDe trial primary outcome was survival free from neurodisability at 2 years’ CA [15]. Secondary outcomes were components of the primary outcome, neonatal morbidity and nutritional intake. The Northern B Health and Disability Ethics Committee gave ethical approval for the study (No 13/NTB/84), the New Zealand (NZ) Newborn Metabolic Screening Unit approved use of amino acid blood spot concentrations, and each participating site had institutional approval through local institutional review processes. Informed written consent was obtained for all participants from their parents or caregivers.

This subgroup analysis comprises 382 babies admitted to the 6 NZ Neonatal Intensive Care Units (NICUs) for whom blood spots were collected for routine newborn metabolic screening both before and after the 5-day intervention period. Amino acids were extracted into methanol and analysed using electrospray ionisation on a triple quadrupole mass spectrometer and quantitated using deuterated internal standards.

Amino acid concentrations are reported at the following time-points: day 0–2 (sample collected day 0 to 2); week 1 (sample collected day 3 to 11); week 2–3 (sample collected day 12 to 24); and week 4 (sample collected day 25 to 35). Although blood samples on day 0–2 were taken before the intervention began and the week 1 sample was taken after the intervention finished and the line was flushed, blood spot amino acid concentration data from the week 1 sample included some unusually high values considered to possibly be secondary to study fluid contamination as the blood sample was withdrawn via the UAC, through which the intervention fluid was delivered. Amino acid concentrations in the ProVIDe dataset were manually coded by the hospital laboratory as ‘ok’, ‘unusual’, ‘insufficient’, ‘inadequate’ or ‘contaminated’ prior to importation for analysis. Values coded as ‘insufficient’, ‘inadequate’ or ‘contaminated’ were excluded from analysis. On day 0–2 one sample was labelled inadequate, 9 were contaminated and 13 were unusual. At week 1, one sample was inadequate, 7 were contaminated and 19 were labelled unusual. All except the day 0–2 inadequate sample were in the intervention group.

Prospectively collected de-identified trial data, including nutritional intake data for the first week after birth, were obtained from ProVIDe trial investigators and downloaded to a Microsoft Excel spreadsheet:

1. Lipid intake (g.Kg.^−1^d^−1^) and brand [SMOFlipid^®^ (Fresenius Kabi), INTRALIPID^®^ (Fresenius Kabi), Lipoplus^®^ (B Braun Medical) or ClinOleic^®^ (Baxter Healthcare)];
2. Amino acid intake (g.Kg.^−1^d^−1^) and brand (TrophAmine^®^ B Braun Medical (2 sites), or Primene^®^ Baxter Healthcare Ltd. (4 sites));
3. ProVIDe RCT intervention (TrophAmine^®^ B Braun Medical) or placebo group.

Clinical outcome assessed at 36 weeks’ CA included severe intraventricular haemorrhage (IVH; grades 3 or 4 as defined by the grading system of Papile et al. [16]), bronchopulmonary dysplasia (BPD; requiring oxygen at 36 weeks’ post-menstrual age or 28 days after birth if born after 32 weeks’ gestation), retinopathy of prematurity (ROP; grades 3 and 4) defined using the International Classification of ROP [17], necrotising enterocolitis (NEC); defined as Bell’s stage ≥2 [18], patent ductus arteriosus (PDA; requiring treatment as diagnosed by echocardiography), early- and late-onset sepsis (early-onset sepsis; occurring ≤48 h after birth, late-onset sepsis; occurring >48 h to 36 weeks; culture-proven sepsis confirmed by positive bacterial culture in cerebrospinal fluid, blood or urine with clinical signs of infection and treated with antibiotics for ≥5 days or for a shorter time if the patient died; probable sepsis defined as sepsis without a positive culture but with clinical signs of sepsis and a plan for ≥5 days of antibiotics to 36 weeks’ postmenstrual age); and death before discharge from neonatal care. Probable sepsis represents a non-definitive outcome and may include non-infective causes of clinical instability that mimic sepsis sufficiently to warrant at least 5 days of antibiotic therapy.

Weight, length, and head circumference data were collected at birth, 28 days, 8 weeks, 36 weeks’ CA and discharge using previously reported methods [14]. At 2 years’ CA, children underwent neurodevelopmental assessment as described previously [14]. Neurodisability was defined as the presence of cerebral palsy, blindness, deafness or developmental delay (Bayley III cognitive, motor or language score < 85).

### Statistical Analysis

De-identified data were imported into Statistical Discovery software JMP^®^ version 16.0 (SAS Institute Inc., Cary, NC, USA) for analysis. Descriptive summaries were presented using mean (SD) for continuous variables and number (%) for categorical variables. For nutrient intake calculations, only babies who survived for 3 or more days after birth were included. Amino acid and lipid intakes, and blood spot amino acid concentrations were analysed by simple linear regression analysis and reported as beta-coefficients. Amino acid and lipid intakes and blood spot amino acid concentrations were categorised into tertiles (for clinical outcome) or quartiles (for neurodevelopment) and analysed against outcomes using odds ratios. As the optimal parenteral intakes and blood spot concentrations of individual amino acids are unknown, for clinical outcomes, we used the lowest tertile as the comparator to increase the subgroup sample size and improve statistical efficiency. For the analysis of neurodevelopmental outcomes, quartiles were used to align with the recommended intakes of these specific amino acids (tyrosine 74 mg.Kg^−1^.day^−1^ [19]; lysine 105 mg.Kg^−1^.day^−1^ [20]; threonine 38 mg.Kg^−1^.day^−1^ [21]; methionine + cysteine mg.Kg^−1^.day^−1^ [22]). Multiple logistic regression adjusted for sex and SGA status was used to calculate the adjusted odds ratios. Given the number of analyses, statistical significance was defined as a *p*-value < 0.01.

## 3. Results

**Figure 3:**
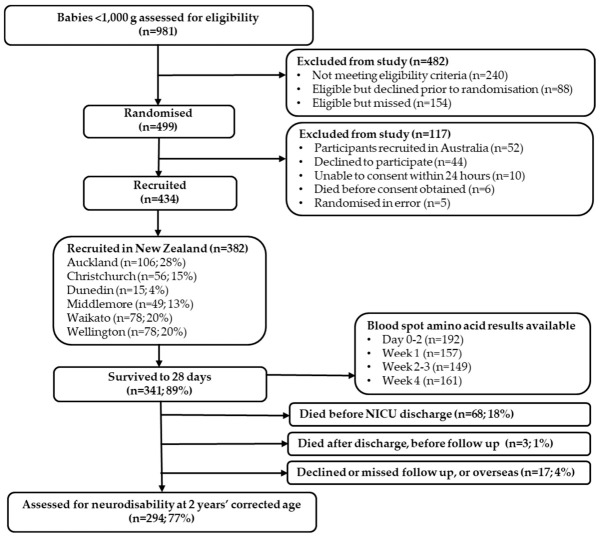
Participant flow diagram.

A total of 382 ELBW babies were recruited at six NZ NICU sites. Of these, 341 babies survived to 28 days, and 294 were assessed at the 2-year follow-up, as shown in Figure 3. Mean (SD) gestational age was 26 weeks and birthweight 782 (134) g, as shown in Table 1.

In this NZ cohort of babies, 40 (11%) had grade 3 or higher IVH and 44 (12%) developed NEC. Of the 320 babies who survived to 36 weeks’ CA, 237 (74%) had BPD, as shown in Supplementary Table S1. Of 294 NZ children who survived to the 2-year follow-up and were assessed for neurodisability, 181 (62%) had no neurodisability while 113 (38%) had mild, moderate or severe neurodisability, as shown in Supplementary Table S1.

### 3.1. Parenteral Amino Acid Intakes

In week 1, there was substantial variation in amino acid intakes, as shown in Table 2.

**Figure 4:**
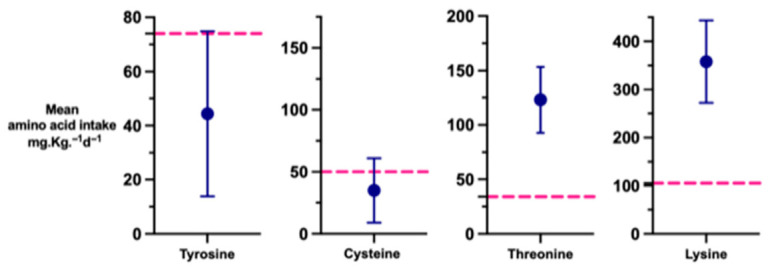
Week 1 mean amino acid intakes in the ProVIDe cohort compared with recommended intakes. Blue dots represent the mean intake and error bars indicate standard deviation (SD). The pink dotted horizontal line denotes the recommended intake for each amino acid: tyrosine 74 mg.Kg^−1^.day^−1^ [19]; lysine 105 mg.Kg^−1^.day^−1^ [20]; threonine 38 mg.Kg^−1^.day^−1^ [21]; cysteine 50 to 75 mg.Kg^−1^.day^−1^ [23].

Mean intakes exceeded international recommendations for lysine and threonine, as shown in Figure 4. In contrast, mean tyrosine intake in week 1 was well below the current recommendation of 74 mg.Kg^−1^.day^−1^ [19,20,21,22] and mean cystine intake was also below the recommendation of 50 to 75 mg.Kg^−1^.day^−1^ [23].

### 3.2. Blood Spot Amino Acids Concentrations

**Figure 5:**
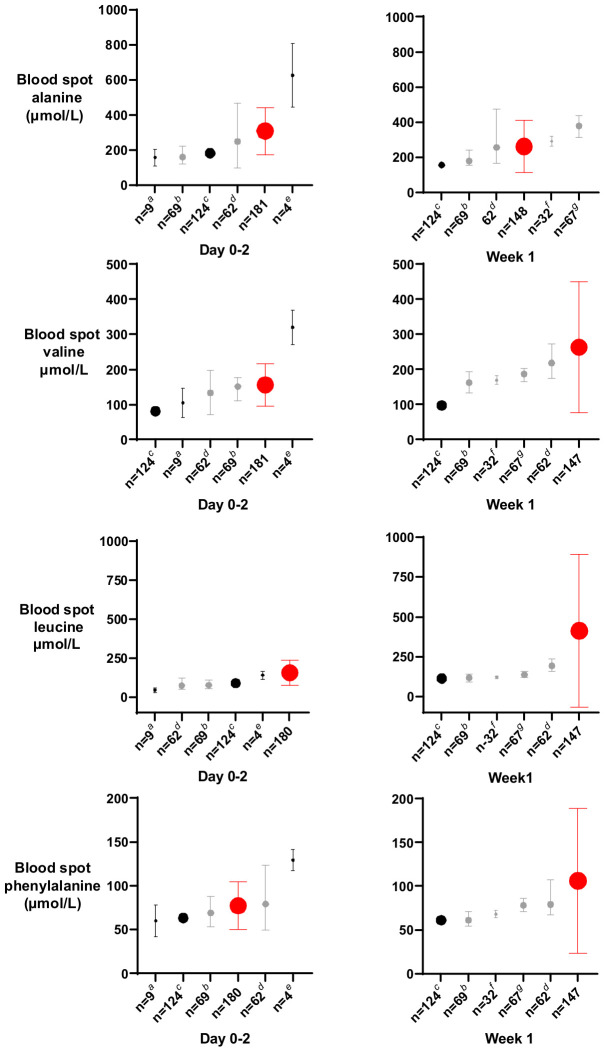

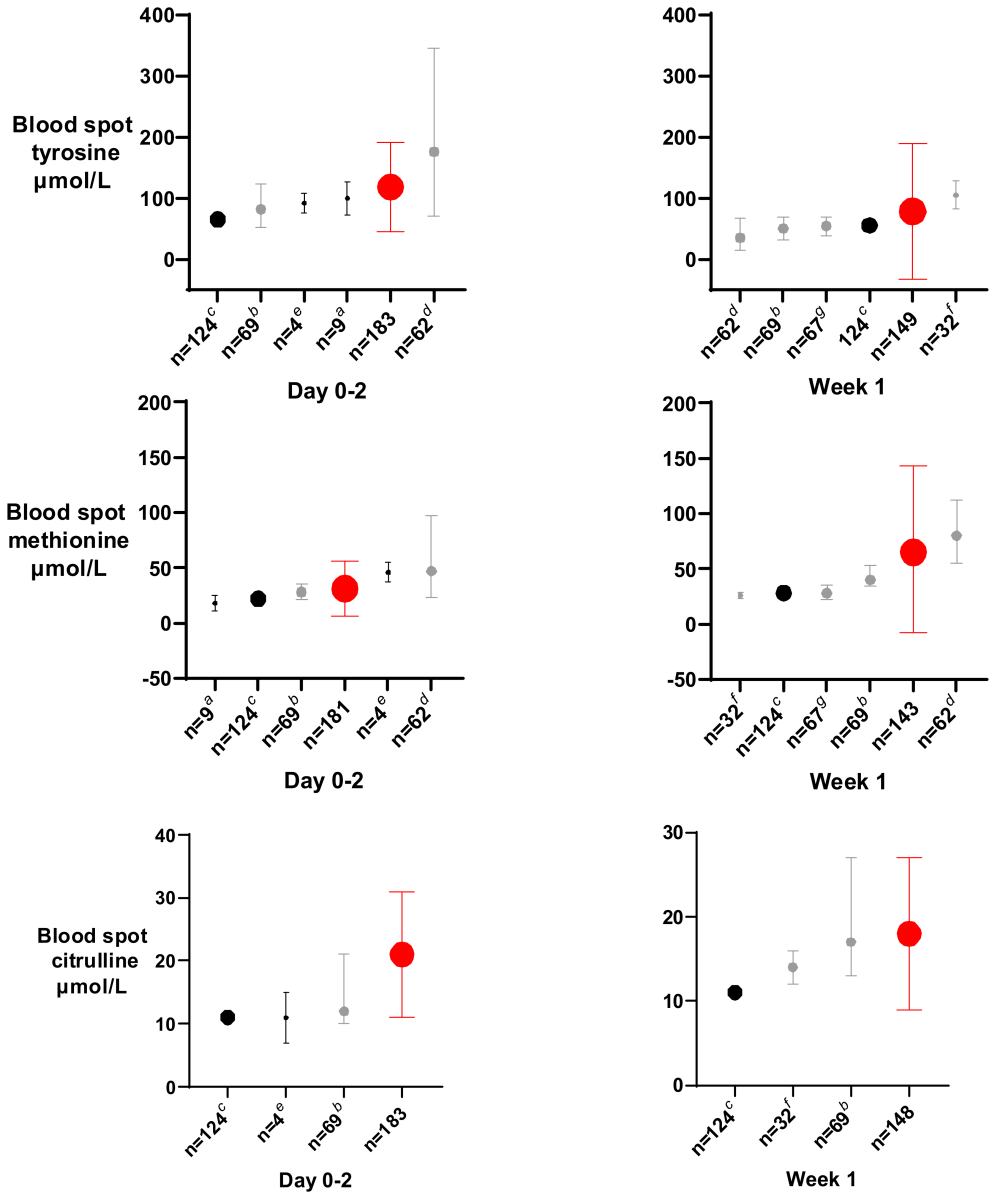
Blood spot/plasma amino acid concentrations on day 0–2 and week 1 in the ProVIDe trial and other studies of preterm babies. Size of circle represents sample size. Red circle represents the ProVIDe trial mean (SD). Black circle represents mean and grey circle represents median. ^a^ [25] mean (SD). ^b^ [26] median (IQR). ^c^ [27] mean. ^d^ [11] median (10th-90th percentile). ^e^ [28] mean (SD). ^f^ [29] median (SD). ^g^ [30] median (IQR).

Blood spot amino acid concentrations on day 0–2 were similar to those reported for preterm and ELBW babies by others at the same postnatal age [11,24], but by week 1 were higher than in previous reports, as shown in Figure 5 and Supplementary Table S2.

**Figure 6:**
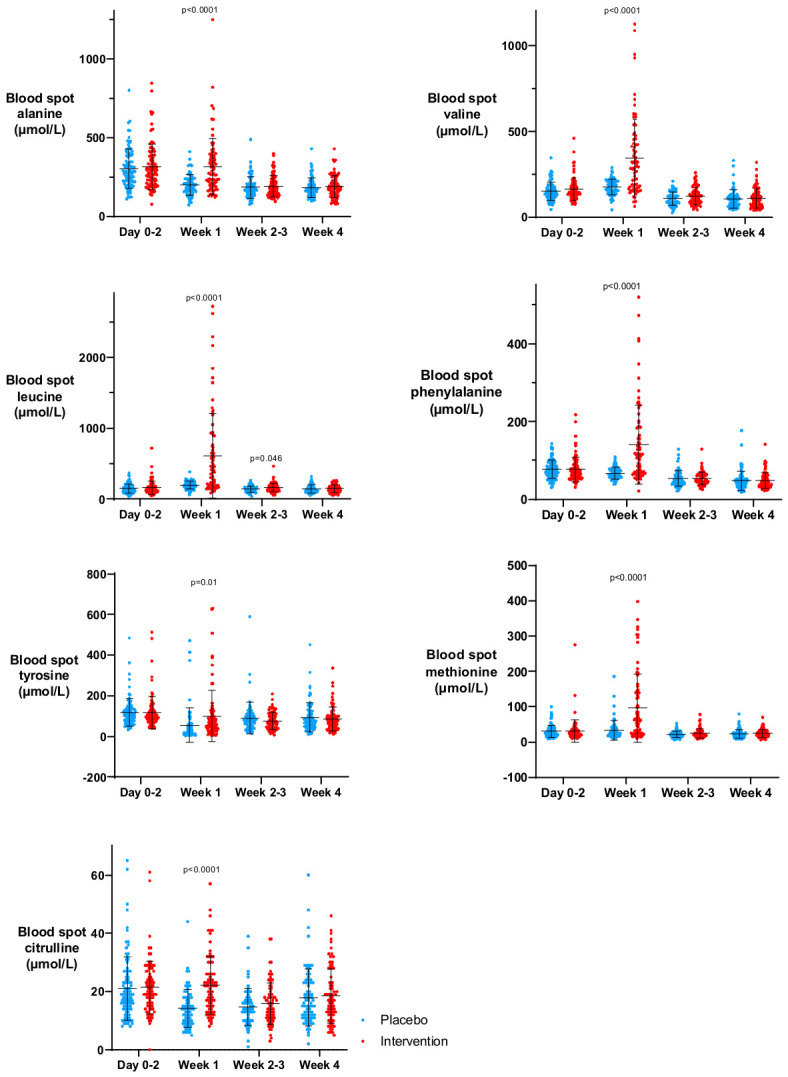
Mean blood spot amino acid concentration on day 0–2, week 1, week 2–3 and week 4, by placebo (blue) and intervention (red) groups. Dots represent data from individual babies. The horizontal line represents the mean, and the whiskers show the standard deviation. The appearance of negative values reflects the use of mean ± standard deviation to summarise right-skewed distributions, where the standard deviation exceeds the mean. As amino acid concentrations are bounded at zero, these negative values are not observed data but a statistical artefact of the summary method.

At week 1, mean blood spot amino acid concentrations were significantly higher in the ProVIDe RCT intervention group vs. the placebo group for each amino acid, but these differences were not seen at any other time point, as shown in Figure 6. Blood spot amino acid concentrations decreased between day 0–2 and week 4 for several amino acids. Week 1 blood spot amino acid concentrations were positively associated with amino acid intakes in the first week after birth for all amino acids, and most strongly for tyrosine, as shown in Supplementary Figure S1.

### 3.3. Clinical Outcome and Amino Acid and Lipid Intakes

#### 3.3.1. Bronchopulmonary Dysplasia

Babies with week 1 intakes in the highest tertile for tyrosine and proline had over twice the odds of BPD (tyrosine: OR 2.2 [CI 1.2,3.9] *p* = 0.007; proline: OR 2.3 [CI 1.3, 4.0] *p* = 0.006) when compared with babies with intakes in the lowest tertile, whereas those with cysteine intakes in the highest tertile had half the odds of developing BPD compared with babies in the lowest tertile (OR 0.5 [CI 0.3, 0.8], *p* = 0.009), as shown in Supplementary Table S3.

#### 3.3.2. Probable Sepsis

There was a dose-dependent relationship between higher intakes of tyrosine and proline and increased odds of early-onset probable sepsis, as shown in Supplementary Table S3. Similarly, higher week 1 intakes of five amino acids (leucine, arginine, proline, methionine and tyrosine) were associated with increased odds of late-onset probable sepsis in a dose-dependent manner. In contrast, higher week 1 intakes of glutamic acid, aspartic acid, histidine, cysteine, and taurine were associated with significantly lower odds of early-onset probable sepsis—most notably cysteine (OR 0.1 [CI < 0.1, 0.2], *p* < 0.0001). There were also dose-dependent associations for four amino acids (glutamic acid, aspartic acid, cysteine and taurine) with lower odds of late-onset sepsis, as shown in Supplementary Table S3.

**Figure 7:**
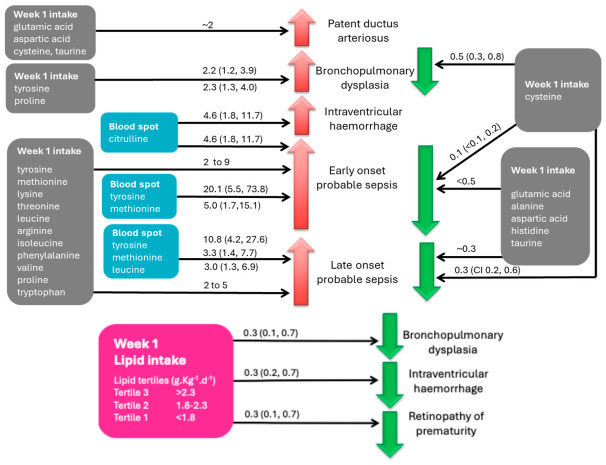
Summary of associations between clinical outcome and parenteral amino acid intake and blood spot fatty acids and lipid intake tertile in week 1. Data are OR (95% CI) adjusted for hospital. Red upward arrows indicate increased odds, and green downward arrows indicate decreased odds of the clinical outcome occurring. Grey boxes relate to amino acid intakes, the pink box relates to lipid emulsion intake, and blue boxes indicate blood spot amino acid concentrations. For further details see Supplementary Tables S3 and S4.

After an adjustment for the baseline brand of parenteral amino acid solution received, most of these associations with sepsis remained significant in a dose-dependent manner, as shown in Table 3 and Supplementary Tables S4 and S5. These associations are summarised in Figure 7.

Higher mean lipid intake in the first week after birth was associated with significantly lower odds of BPD and both early- and late-onset probable sepsis. When adjusted for the hospital, higher mean week 1 parenteral lipid intake was associated only with lower odds of IVH, ROP and BPD, as shown in Table 4 and Figure 7.

### 3.4. Clinical Outcomes and Blood Spot Amino Acid Concentrations

For blood spot amino acid concentrations on day 0 to 2, there were no associations with clinical outcomes. For week 1 blood spot concentrations, babies with citrulline concentrations in the highest tertile had higher odds of IVH compared to those in the lowest tertile, as shown in Table 5. Babies with week 1 blood spot concentrations of tyrosine, methionine and citrulline in the highest tertile had higher odds of early-onset probable sepsis compared to those in the lowest tertile, and those with week 1 blood spot concentrations of tyrosine and methionine in the highest tertile had higher odds of late-onset probable sepsis, as shown in Table 5 and Figure 7.

### 3.5. Neurodevelopmental Outcomes

We found no associations between week 1 tyrosine, lysine, threonine and methionine + cysteine intakes and Bayley III scores (Supplementary Figure S2), and the odds of neurodisability did not differ according to intake quartile for any amino acid in week 1, as shown in Supplementary Table S6. Similarly, the odds of neurodisability did not differ by quartile of week 1 blood spot amino acids, as shown in Supplementary Table S7.

### 3.6. Differences by Amino Acid or Lipid Brand

In the placebo only group, babies who received TrophAmine as their routine IV amino acid solution had higher odds of early- and late-onset probable sepsis compared with babies who received Primene, Table 6.

The majority (79%) of the babies received SMOFlipid, 18% received ClinOleic, 2% received Intralipid, one baby received Lipoplus, and one died before receiving a lipid emulsion.

When compared to babies receiving ClinOleic, babies who received SMOFlipid had lower odds of PDA (OR 0.5 [CI 0.3,0.8], *p* = 0.004) and higher odds of developing early-onset probable sepsis (OR 5.2 [CI 2.3,11.6] *p* = 0.004). The presence of fish oil compared with no fish oil in IV lipid emulsion was also associated with lower odds of PDA (OR 0.54 [CI 0.35,0.84], *p* = 0.005) and higher odds of early-onset probable sepsis (early: OR 3.0 [CI 1.7,3.4] *p* = 0.004). There were no differences in neurodevelopment by amino acid or lipid brand.

## 4. Discussion

In this multicentre cohort of ELBW babies, we found associations between higher lipid intake in the first week after birth and lower incidences of IVH, BPD and ROP. These findings challenge earlier studies that suggested early parenteral lipid use in preterm babies was harmful, leading to cautious use for decades [31,32]. However, we acknowledge these earlier studies were in different gestational age groups and used different lipid formulations. Recent research supports the safety of early higher lipid intake, although studies on higher early lipid in ELBW babies remain limited [33]. Although ESPGHAN guidelines recommend lipid intakes up to 4 g.Kg^−1^.d^−1^ in the first two days after birth, many neonatal units still begin at 0.5–1 g.Kg^−1^.d^−1^ and increase gradually [34]. Our findings support earlier and higher lipid intakes, even for ELBW babies.

The BPD incidence (74%) in this cohort is similar to the published rates of 72% at 26 weeks of gestational age in the Australian and New Zealand Neonatal Network, and within their range of 46% at 28 weeks to 95% at 24 weeks of gestational age [35]. Preterm babies often show elevated inflammatory cytokines, which are associated with morbidities such as BPD and IVH [36]. While omega-3 fatty acids in modern lipid emulsions may reduce inflammation [37], a systematic review of 22 studies including 3781 preterm infants found no evidence that these emulsions prevent BPD [38]. Our study adds to this literature by examining the timing: we observed that higher lipid intakes in the first week were associated with 64% lower odds of BPD and lower odds of ROP. These findings suggest that early provision of lipids, rather than the type of emulsion alone, may be key. Potential mechanisms include reduced oxidative stress [39,40], improved retinal arachidonic acid and docosahexaenoic acid availability [33], and provision of energy and vitamin A, all of which could influence BPD and ROP risk [41,42]. In New Zealand, fat-soluble vitamins are supplied via lipid emulsions.

We also found positive associations between individual amino acid intakes and the incidence of BPD, PDA and probable sepsis. A plausible mechanism is that elevated serum concentrations of specific amino acids (e.g., tyrosine, proline) reflect a mismatch between high amino acid provision and immature metabolic capacity in extremely preterm infants [11]. This can lead to the accumulation of unmetabolised substrates, contributing to oxidative stress (particularly for tyrosine), impaired tissue remodelling (proline) and disrupted vascular regulation. These processes are central to the pathogenesis of BPD and PDA [43]. In addition, excess amino acid delivery may promote an anabolic drive with relative electrolyte depletion and hypophosphataemia, consistent with neonatal refeeding syndrome, which may present clinically as the instability labelled as “sepsis.”

Recommended intakes of some amino acids are included in the ESPGHAN 2018 guidelines [23], although the tyrosine recommendation was based on a study in term-born, not ELBW, babies [19]. In our cohort, few babies received the recommended tyrosine intake (74 mg.Kg^−1^.d^−1^), yet those in the highest intake tertile (>49 mg.Kg^−1^.d^−1^) had higher odds of BPD compared with babies with intakes in the lowest tertile. Furthermore, lysine, threonine, and methionine + cysteine intakes were 2–3 times higher than ESPGHAN recommendations. Higher lysine intake correlated with worse outcomes, while higher cysteine intake was associated with lower BPD risk. The magnitude of the observed protective association for cysteine is interesting but may reflect residual confounding, collinearity with specific parenteral amino acid formulations or differences in clinical practice. However, cysteine is a conditionally essential amino acid in preterm babies and the rate-limiting substrate for glutathione synthesis, a key intracellular antioxidant. Extremely preterm infants have limited capacity to synthesise cysteine from methionine, predisposing them to glutathione depletion and oxidative stress. Several studies show that cysteine or *N*-acetylcysteine supplementation increases glutathione concentrations and may attenuate oxidative injury. As oxidative stress is central to the pathogenesis of BPD, improving glutathione status through adequate cysteine provision is biologically plausible as a protective strategy, although clinical outcome data remain inconsistent [44]. These findings suggest that modifying amino acid formulations to lower lysine and increase cysteine may improve outcomes and warrants further study.

Blood spot amino acid concentrations in our cohort (receiving up to 5 g.Kg^−1^.d^−1^ of parenteral amino acid) were similar to previous studies of babies receiving up to 4 g.Kg^−1^.d^−1^ of parenteral amino acid [11,24]. Certain blood spot amino acids (e.g., tyrosine, methionine, proline and leucine) were consistently associated with both early and late probable sepsis, though not with culture-proven sepsis. These amino acids are more abundant in TrophAmine, which was the intervention amino acid for the ProVIDe RCT. In contrast, amino acids associated with lower sepsis risk (glutamic acid, aspartic acid, cysteine, taurine) are higher in Primene. This suggests formulation differences may influence outcomes and should be further explored.

Probable sepsis reflects a composite of clinical signs and the clinical decision to treat with at least five days of antibiotics rather than confirmed infection. As the ProVIDe trial definition of probable sepsis was “clinical signs of infection and antibiotic therapy for at least five days,” these symptoms might reflect refeeding syndrome rather than infection, although the inclusion of at least five days of antibiotic therapy somewhat mitigates that possibility. The associations with probable rather than culture-proven sepsis are likely simply due to the event rate [15]. Neonatal refeeding syndrome is not well-defined [45], but a secondary analysis in the same ProVIDe cohort showed that higher amino acid intake was associated with refeeding syndrome (mean (SD) 3.3 (0.8) vs. 3.0 (0.7) g.Kg^−1^.d^−1^, *p* = 0.001) [10].

While specific amino acids such as cysteine, arginine, taurine and tyrosine have recognised roles in antioxidant defence, cerebral perfusion, neurotransmitter synthesis and neuronal development, evidence linking individual amino acids to improved neurodevelopmental outcomes remains limited and inconsistent. Studies more consistently demonstrate associations with total amino acid or protein intake rather than isolated amino acids [13,23,46]. This likely reflects the interdependent nature of amino acid metabolism and the importance of overall protein and energy adequacy in supporting neurodevelopment, which may explain the absence of associations observed with individual amino acid intakes and neurodevelopment in our cohort. Given the correlation between amino acid intake and blood spot concentrations, associations observed with blood spot concentrations are likely not independent of intake and may represent downstream markers of exposure rather than causal effects, which should be considered when interpreting these findings.

The neurodisability rate in our cohort is similar to those reported in recent studies, which range from 33 to 41% [47,48,49], although the rate is lower than in other reports of 50 to 66% in ELBW and extremely preterm studies [50,51,52,53]. The absence of associations with neurodevelopment at two years may have been due to the study being underpowered for this outcome, the follow-up at two years rather than later, or that we may not have captured the most biologically relevant amino acids or exposure windows influencing longer-term neurodevelopment; however, it is also possible that no true association exists, particularly given that we found severe hypophosphataemia was associated with increased neurodisability at two years (aOR 2.31 (95% CI 1.22 to 4.35), *p* = 0.01) in this cohort using a similar analytical approach [54].

### Strengths and Limitations

The strengths of this study include its large, multicentre design, prospective data collection, standardised protocols within an RCT and minimal loss to follow-up (5% at 2-year follow-up). It is one of the few studies to examine a wide range of outcomes in ELBW babies exposed to different parenteral nutrition regimens.

The limitations include the blood sample collection time point definitions, which are clinically meaningful but ranged from “day 3 to 11” for “week 1”. Our use of the lowest tertile as the reference group may introduce bias if this category reflects relatively inadequate intake or greater illness severity. Confounding by indication is a potential concern, but previous analyses in the ProVIDe cohort demonstrated that nutrient intakes were primarily determined by the hospital site rather than clinical severity, reducing the likelihood of this bias [9]. While a more stringent significance threshold (*p* < 0.01) was applied to partially account for multiple comparisons, no formal adjustment for multiplicity was undertaken; therefore, given the large number of analyses, these findings should be considered exploratory and interpreted with caution due to the potential for false positive results. The cohort was drawn exclusively from New Zealand, which may limit generalisability to settings with different parenteral nutrition practices. Finally, blood spot amino acid concentrations represent a proxy for plasma concentrations and may not reflect tissue-level amino acid status. While findings from this research cannot infer causality, they provide valuable insights for the composition and prescription of early parenteral nutrition for ELBW babies.

## 5. Conclusions

Higher early parenteral lipid intake is associated with lower morbidity in neonatal care, supporting ESPGHAN’s 2018 guidelines [23] of up to 4 g.Kg^−1^.d^−1^ in the first two days after birth. Further research is needed to define the optimal composition and dosage of amino acid and lipid solutions to improve outcomes in this vulnerable population.

## Figures and Tables

**Table 1:** Baseline characteristics.

Characteristic	Overall ProVIDe RCT *n* = 434 ^a^	NZ-Born ProVIDe Cohort in This Study *n* = 382 ^b^	Assessed for Neurodisability *n* = 294 ^c^
**Neonatal factors**			
Gestational age at birth (weeks + days)	25^+6^ (1^+4^)	26^+3^ (2^+2^)	26^+1^ (1^+5^)
Birth weight (g)	775 (135)	782 (134)	797 (130)
Birth length (cm)	32.8 (2.4)	32.9 (2.3)	33.2 (2.2)
Birth head circumference (cm)	23.3 (1.6)	23.3 (1.5)	23.5 (1.5)
Birth weight z-score	−0.1 (0.9)	−0.04 (0.9)	−0.07 (0.9)
Birth length z-score	−0.1 (1.0)	−0.09 (1.0)	−0.08 (1.0)
Birth head circumference z-score	0.0 (0.9)	0.02 (0.9)	0.01 (0.9)
Male	212 (49)	180 (47)	129 (44)
Singleton	340 (78)	309 (81)	243 (83)
**Maternal factors**			
Caesarean section	234 (54)	203 (53)	171 (58)
Maternal diabetes	24 (6)	22 (6)	18 (6)
Antenatal corticosteroid use	410 (94)	362 (95)	281 (96)
*Education* ^d^			
Primary school	3 (1)	2 (<1)	2 (<1)
Lower secondary school	64 (15)	50 (13)	34 (12)
Upper secondary school	109 (26)	88 (24)	66 (23)
Post-secondary non-tertiary	114 (27)	112 (30)	92 (32)
University	134 (31)	120 (32)	98 (34)
*Ethnicity*			
European	210 (48)	169 (44)	134 (46)
Māori	102 (24)	102 (27)	77 (26)
Asian	73 (17)	68 (18)	54 (18)
Pacific Island	40 (9)	40 (11)	26 (9)
Other	9 (2)	0.8 (<1)	3 (1)

Data are mean (SD) or n (%) as appropriate. ^a^ All babies in the ProVIDe RCT. ^b^ Babies in the New Zealand ProVIDe cohort in this study. ^c^ Babies in this study cohort who were assessed for neurodisability at 2 years’ CA. ^d^ Missing data *n* = 10.

**Table 2:** Week 1 amino acid intakes in the NZ ProVIDe cohort.

Amino Acid	Week 1 Intake Mean (SD) mg.Kg^−1^.day^−1^
**Lysine**	358 (86)
**Glutamic acid**	233 (80)
**Leucine**	374 (110)
**Arginine**	318 (95)
**Alanine**	208 (55)
**Valine**	239 (56)
**Isoleucine**	232 (61)
**Aspartic acid**	143 (46)
**Phenylalanine**	140 (35)
**Glycine**	118 (27)
**Serine**	121 (28)
**Histidine**	96 (27)
**Threonine**	123 (30)
**Proline**	153 (68)
**Methionine**	90 (27)
**Tryptophan**	62 (14)
**Cysteine**	35 (26)
**Taurine**	14 (5)
**Tyrosine**	44 (30)

**Table 3:** Significant odds ratios for probable sepsis by amino acid intake tertile adjusted for baseline amino acid brand.

Amino Acid	Tertile (mg.kg^−1^.d^−1^)	Odds Ratio Early-Onset Probable Sepsis	*p*-Value	Odds Ratio Late-Onset Probable Sepsis	*p*-Value
Leucine	<307.6	Reference	-	Reference	-
	307.6–410.4	1.23 (0.59–2.55)	0.58	1.79 (1.00–3.22)	0.05
	>410.4	2.75 (1.36–5.57)	**0.005**	2.47 (1.33–4.57)	**0.004**
Arginine	<258.3	Reference	-	Reference	-
	258.3–348.1	1.19 (0.57–2.47)	0.65	1.81 (1.01–3.24)	0.05
	>348.1	2.86 (1.40–5.84)	**0.004**	2.46 (1.32–4.58)	**0.004**
Methionine	<73.9	Reference	-	Reference	-
	73.9–98.9	1.23 (0.59–2.55)	0.58	1.79 (1.00–3.22)	0.05
	>98.9	2.75 (1.36–5.57)	**0.005**	2.47 (1.33–4.57)	**0.004**
Proline	<107.9	Reference	-	Reference	-
	107.9–168.6	5.52 (2.03–14.99)	**0.0008**	1.24 (0.58–2.64)	0.57
	>168.6	7.92 (2.79–22.48)	**0.0001**	1.77 (0.80–3.92)	0.16
Tyrosine	<21	Reference	-	Reference	-
	21–49.3	1.95 (0.95–4.02)	0.07	2.56 (1.54–4.26)	**0.0003**
	>49.3	6.55 (2.69–15.96)	**<0.0001**	6.06 (1.94–8.51)	**0.0002**
Aspartic acid	<121	Reference	-	Reference	-
	121–167.9	0.43 (0.25–0.77)	**0.004**	0.50 (0.30–0.85)	0.01
	>167.9	0.37 (0.18–0.79)	0.01	0.66 (0.35–1.23)	0.19
Cysteine	<8.6	Reference	-	Reference	-
	8.6–50.9	0.15 (0.05–0.46)	**0.0009**	0.30 (0.13–0.68)	**0.004**
	>50.9	0.08 (0.03–0.23)	**<0.0001**	0.33 (0.16–0.67)	**0.002**
Glutamic acid	<191.1	Reference	-	Reference	-
	191.1–278.3	0.51 (0.29–0.92)	0.02	0.46 (0.26–0.79)	**0.005**
	>278.3	0.41 (0.19–0.88)	0.02	0.57 (0.30–1.08)	0.08

Data are presented as odds ratio (95% CI). Significant *p* value < 0.01 shown in bold.

**Table 4:** Odds ratios of clinical outcomes by week 1 lipid intake tertile.

Clinical Outcome	Mean Week 1 Lipid Intake Tertile (g.Kg^−1^.d^−1^)	Unadjusted		Adjusted	
		Odds Ratio (95% CI)	*p*-Value	Odds Ratio (95% CI)	*p*-Value
Intraventricular haemorrhage	<1.82	Reference	-	Reference	-
	1.82–2.29	0.58 (0.36–0.94)	0.03	0.43 (0.24–0.78)	**0.005**
	>2.29	0.47 (0.28–0.78)	**0.003**	0.33 (0.16–0.66)	**0.002**
Bronchopulmonary dysplasia	<1.82	Reference	-	Reference	-
	1.82–2.29	0.46 (0.24–0.86)	0.02	0.51 (0.25–1.04)	0.06
	>2.29	0.36 (0.19–0.67)	**0.001**	0.31 (0.13–0.73)	**0.007**
Retinopathy of prematurity	<1.82	Reference	-	Reference	-
	1.82–2.29	0.62 (0.32–1.20)	0.16	0.57 (0.27–1.23)	0.15
	>2.29	0.62 (0.32–1.20)	0.16	0.29 (0.12–0.72)	**0.008**
Necrotising enterocolitis	<1.82	Reference	-	Reference	-
	1.82–2.29	0.77 (0.37–1.61)	0.48	0.61 (0.26–1.45)	0.26
	>2.29	1.22 (0.62–2.41)	0.57	0.77 (0.29–2.08)	0.61
Patent ductus arteriosus	<1.82	Reference	-	Reference	-
	1.82–2.29	0.71 (0.44–1.15)	0.17	0.55 (0.30–0.99)	0.047
	>2.29	1.12 (0.69–1.81)	0.64	0.42 (0.20–0.92)	0.03
Early onset sepsis—culture proven	<1.82	Reference	-	Reference	-
	1.82–2.29	0.89 (0.18–4.48)	0.89	0.72 (0.12–4.33)	0.72
	>2.29	2.18 (0.55–8.61)	0.27	0.98 (0.13–7.16)	0.99
Early onset sepsis—probable	<1.82	Reference	-	Reference	-
	1.82–2.29	0.39 (0.24–0.65)	**0.0002**	0.56 (0.25–1.23)	0.15
	>2.29	0.04 (0.02–0.11)	**<0.0001**	0.23 (0.06–0.85)	0.03
Late onset sepsis—culture proven	<1.82	Reference	-	Reference	-
	1.82–2.29	0.57 (0.35–0.94)	0.03	0.56 (0.32–0.98)	0.04
	>2.29	0.61 (0.37–0.99)	0.049	0.47 (0.23–0.95)	0.03
Late onset sepsis—probable	>1.82	Reference	-	Reference	-
	1.82–2.29	0.70 (0.44–1.13)	0.14	0.88 (0.47–1.65)	0.69
	>2.29	0.31 (0.19–0.50)	**<0.0001**	0.90 (0.41–1.94)	0.78
Death before discharge	<1.82	Reference	-	Reference	-
	1.82–2.29	1.02 (0.57–1.86)	0.93	0.72 (0.36–1.44)	0.35
	>2.29	0.70 (0.37–1.33)	0.28	0.37 (0.15–0.89)	0.03

Data are odds ratio (95% CI). Significant *p*-value < 0.01 shown in bold.

**Table 5:** Odds ratios of clinical outcomes by blood spot amino acid tertile in week 1.

Clinical Outcome	Tertile	Odds Ratio	*p*-Value
**Intraventricular haemorrhage**			
**Alanine**	<190	Reference	-
	190–264	1.45 (0.62–3.39)	0.39
	>264	1.29 (0.55–3.02)	0.56
**Valine**	<165	Reference	-
	165–239	1.91 (0.81–4.48)	0.14
	>239	1.22 (0.51–2.94)	0.65
**Leucine**	<185	Reference	-
	185–278	2.31 (0.98–5.48)	0.06
	>278	1.36 (0.56–3.31)	0.50
**Phenylalanine**	<65	Reference	-
	65–93	2.00 (0.84–4.75)	0.12
	>93	1.45 (0.60–3.52)	0.41
**Tyrosine**	<30	Reference	-
	30–67	1.69 (0.72–3.95)	0.23
	>67	1.56 (0.66–3.71)	0.31
**Methionine**	<23	Reference	-
	23–49	1.31 (0.54–3.14)	0.55
	>49	1.71 (0.72–4.06)	0.22
**Citrulline**	<14	Reference	-
	14–21	3.40 (1.32–8.79)	0.01
	>21	4.55 (1.77–11.69)	**0.002**
**Bronchopulmonary dysplasia**			
**Alanine**	<190	Reference	-
	190–264	0.80 (0.28–2.27)	0.68
	>264	0.49 (0.18–1.32)	0.16
**Valine**	<165	Reference	-
	165–239	1.68 (0.61–4.60)	0.32
	>239	1.26 (0.49–3.22)	0.63
**Leucine**	<185	Reference	-
	185–278	0.97 (0.36–2.60)	0.95
	>278	0.94 (0.36–2.47)	0.90
**Phenylalanine**	<65	Reference	-
	65–93	1.47 (0.54–3.97)	0.45
	>93	1.20 (0.46–3.12)	0.71
**Tyrosine**	<30	Reference	-
	30–67	1.83 (0.70–4.80)	0.22
	>67	2.19 (0.82–5.87)	0.12
**Methionine**	<23	Reference	-
	23–49	1.02 (0.39–2.67)	0.97
	>49	1.38 (0.51–3.72)	0.53
**Citrulline**	<14	Reference	-
	14–21	0.61 (0.24–1.58)	0.31
	>21	1.14 (0.40–3.25)	0.80
**Retinopathy of prematurity**			
**Alanine**	<190	Reference	-
	190–264	0.52 (0.18–1.50)	0.23
	>264	0.37 (0.12–1.18)	0.09
**Valine**	<165	Reference	-
	165–239	0.93 (0.34–2.56)	0.88
	>239	0.35 (0.10–1.22)	0.10
**Leucine**	<185	Reference	-
	185–278	0.93 (0.34–2.56)	0.88
	>278	0.35 (0.10–1.22)	0.10
**Phenylalanine**	<65	Reference	-
	65–93	0.61 (0.21–1.78)	0.36
	>93	0.52 (0.17–1.59)	0.25
**Tyrosine**	<30	Reference	-
	30–67	0.94 (0.33–2.70)	0.91
	>67	0.68 (0.22–2.11)	0.51
**Methionine**	<23	Reference	-
	23–49	1.00 (0.34–2.96)	1.00
	>49	0.88 (0.29–2.67)	0.81
**Citrulline**	<14	Reference	-
	14–21	0.49 (0.17–1.40)	0.18
	>21	0.29 (0.09–0.98)	0.05
**Necrotising enterocolitis**			
**Alanine**	<190	Reference	-
	190–264	0.40 (0.11–1.38)	0.15
	>264	0.72 (0.25–2.13)	0.56
**Valine**	<165	Reference	-
	165–239	0.25 (0.81–7.99)	0.11
	>239	0.78 (0.20–3.11)	0.73
**Leucine**	<185	Reference	-
	185–278	1.61 (0.53–4.94)	0.40
	>278	0.81 (0.23–2.87)	0.75
**Phenylalanine**	<65	Reference	-
	65–93	2.15 (0.68–6.84)	0.19
	>93	0.98 (0.26–3.62)	0.97
**Tyrosine**	<30	Reference	-
	30–67	1.67 (0.51–5.52)	0.40
	>67	1.54 (0.45–5.22)	0.49
**Methionine**	<23	Reference	-
	23–49	0.98 (0.31–3.03)	0.97
	>49	0.82 (0.25–2.64)	0.73
**Citrulline**	<14	Reference	-
	14–21	0.48 (0.12–1.63)	0.24
	>21	1.08 (0.36–3.18)	0.89
**Patent ductus arteriosus**			
**Alanine**	<190	Reference	-
	190–264	1.40 (0.63–3.12)	0.41
	>264	0.96 (0.43–2.14)	0.93
**Valine**	<165	Reference	-
	165–239	1.35 (0.60–3.02)	0.47
	>239	1.10 (0.49–2.45)	0.82
**Leucine**	<185	Reference	-
	185–278	0.74 (0.33–1.67)	0.47
	>278	0.72 (0.32–1.60)	0.42
**Phenylalanine**	<65	Reference	-
	65–93	0.81 (0.36–1.82)	0.61
	>93	1.09 (0.49–2.42)	0.84
**Tyrosine**	<30	Reference	-
	30–67	1.05 (0.48–2.32)	0.90
	>67	1.34 (0.60–3.01)	0.47
**Methionine**	<23	Reference	-
	23–49	0.92 (0.41–2.10)	0.85
	>49	1.14 (0.51–2.58)	0.75
**Citrulline**	<14	Reference	-
	14–21	0.72 (0.32–1.60)	0.42
	>21	1.29 (0.58–2.88)	0.53
**Early onset sepsis—culture proven**			
**Alanine**	<190	Reference	-
	190–264	1.00 (0.06–16.45)	1.00
	>264	2.00 (0.18–22.80)	0.58
**Valine**	<165	Reference	-
	165–239	1.00 (0.06–16.45)	1.00
	>239	2.04 (0.18–23.29)	0.57
**Leucine**	<185	Reference	-
	185–278	1.00 (0.06–16.45)	1.00
	>278	2.04 (0.18–23.29)	0.57
**Phenylalanine**	<65	Reference	-
	65–93	<0.001 (not estimable)	0.99
	>93	0.98 (0.13–7.24)	0.98
**Tyrosine**	<30	Reference	-
	30–67	0.48 (0.04–5.47)	0.55
	>67	0.51 (0.04–5.82)	0.59
**Methionine**	<23	Reference	-
	23–49	0.98 (0.06–16.12)	0.99
	>49	2.00 (0.18–22.83)	0.58
**Citrulline**	<14	Reference	-
	14–21	0.51 (0.04–5.82)	0.59
	>21	0.52 (0.05–5.94)	0.60
**Early onset sepsis—probable**			
**Alanine**	<190	Reference	-
	190–264	0.72 (0.29–1.80)	0.49
	>264	0.88 (0.36–2.13)	0.77
**Valine**	<165	Reference	-
	165–239	2.05 (0.77–5.46)	0.15
	>239	2.26 (0.86–5.97)	0.10
**Leucine**	<185	Reference	-
	185–278	3.47 (1.23–9.85)	0.02
	>278	3.16 (1.11–9.02)	0.03
**Phenylalanine**	<65	Reference	-
	65–93	2.28 (0.83–6.26)	0.11
	>93	2.84 (1.05–7.72)	0.04
**Tyrosine**	<30	Reference	-
	30–67	3.82 (0.98–14.84)	0.05
	>67	20.14 (5.50–73.84)	**<0.0001**
**Methionine**	<23	Reference	-
	23–49	2.80 (0.90–8.70)	0.08
	>49	5.04 (1.68–15.08)	**0.004**
**Citrulline**	<14	Reference	-
	14–21	1.05 (0.36–3.06)	0.93
	>21	4.55 (1.77–11.69)	**0.002**
**Late onset sepsis—culture proven**			
**Alanine**	<190	Reference	-
	190–264	2.38 (0.99–5.75)	0.05
	>264	1.48 (0.60–3.65)	0.39
**Valine**	<165	Reference	-
	165–239	1.91 (0.81–4.48)	0.14
	>239	1.00 (0.41–2.45)	1.00
**Leucine**	<185	Reference	-
	185–278	2.38 (0.99–5.74)	0.05
	>278	1.52 (0.62–3.77)	0.36
**Phenylalanine**	<65	Reference	-
	65–93	1.84 (0.77–4.38)	0.17
	>93	1.32 (0.54–3.23)	0.54
**Tyrosine**	<30	Reference	-
	30–67	2.58 (1.06–6.30)	0.04
	>67	2.00 (080–5.00)	0.14
**Methionine**	<23	Reference	-
	23–49	1.91 (0.80–4.58)	0.15
	>49	1.20 (0.49–2.97)	0.69
**Citrulline**	<14	Reference	-
	14–21	1.70 (0.72–4.00)	0.23
	>21	1.33 (0.55–3.19)	0.53
**Late onset sepsis—probable**			
**Alanine**	<190	Reference	-
	190–264	1.28 (0.58–2.83)	0.54
	>264	1.05 (0.47–2.31)	0.91
**Valine**	<165	Reference	-
	165–239	1.79 (0.80–4.02)	0.16
	>239	1.95 (0.87–4.37)	0.11
**Leucine**	<185	Reference	-
	185–278	3.02 (1.32–6.93)	**0.009**
	>278	2.56 (1.12–5.86)	0.03
**Phenylalanine**	<65	Reference	-
	65–93	2.58 (1.13–5.90)	0.02
	>93	2.70 (1.18–6.19)	0.02
**Tyrosine**	<30	Reference	-
	30–67	4.16 (1.72–10.07)	**0.002**
	>67	10.77 (4.20–27.60)	**<0.0001**
**Methionine**	<23	Reference	-
	23–49	2.56 (1.10–5.96)	0.03
	>49	3.30 (1.41–7.71)	**0.006**
**Citrulline**	<14	Reference	-
	14–21	1.26 (0.57–2.82)	0.57
	>21	2.81 (1.24–6.34)	0.01
**Death before discharge**			
**Alanine**	<190	Reference	-
	190–264	0.70 (0.22–2.19)	0.54
	>264	0.85 (0.28–2.57)	0.78
**Valine**	<165	Reference	-
	165–239	1.84 (0.61–5.55)	0.28
	>239	0.81 (0.23–2.87)	0.75
**Leucine**	<185	Reference	-
	185–278	2.32 (0.73–7.37)	0.16
	>278	1.29 (0.37–4.54)	0.69
**Phenylalanine**	<65	Reference	-
	65–93	1.32 (0.45–3.88)	0.62
	>93	0.70 (0.20–2.38)	0.56
**Tyrosine**	<30	Reference	-
	30–67	1.83 (0.61–5.50)	0.28
	>67	0.89 (0.25–3.16)	0.86
**Methionine**	<23	Reference	-
	23–49	2.04 (0.63–6.64)	0.24
	>49	1.47 (0.43–5.01)	0.54
**Citrulline**	<14	Reference	-
	14–21	0.71 (0.21–2.43)	0.59
	>21	1.45 (0.49–4.28)	0.50

Data are presented as odds ratio (95% CI). Significant *p*-value < 0.01 shown in bold.

**Table 6:** Odds ratios of clinical outcome by amino acid brand in the placebo group.

Clinical Outcome	Amino Acid Brand		OR (95% CI)	*p*-Value
	Primene	TrophAmine		
Intraventricular haemorrhage	38/116 (32.8%)	27/92 (29.4%)	0.85 (0.47–1.54)	0.60
Bronchopulmonary dysplasia	65/99 (65.7%)	61/82 (74.4%)	1.52 (0.80–2.90)	0.20
Retinopathy of prematurity	17/104 (16.4%)	13/83 (15.7%)	0.95 (0.43–2.09)	0.90
Necrotising enterocolitis	16/116 (13.8%)	10/92 (10.9%)	0.76 (0.33–1.77)	0.53
Patent ductus arteriosus	48/114 (42.1%)	36/88 (40.9%)	0.95 (0.54–1.67)	0.86
Early onset sepsis—culture proven	5/116 (4.3%)	1/91 (1.1%)	0.25 (0.03–2.15)	0.17
Early onset sepsis—probable	14/116 (12.1%)	37/91 (40.7%)	4.99 (2.48–10.03)	**<0.0001**
Late onset sepsis—culture proven	31/116 (26.7%)	33/91 (36.3%)	1.56 (0.86–2.82)	0.14
Late onset sepsis—probable	32/116 (27.6%)	49/91 (53.9%)	3.06 (1.72–5.47)	**0.0001**
Death before discharge	24/113 (21.2%)	14/93 (15.1%)	0.66 (0.32–1.36)	0.25

Data are presented as number (percent) and odds ratio (95% CI). Significant *p* value < 0.01 shown in bold.

## Data Availability

Data are available upon reasonable request to the Liggins Institute Data Access Committee, subject to appropriate approvals and data access agreements.

## Supplementary Materials

The following supporting information can be downloaded at https://www.mdpi.com/article/10.3390/nu18101517/s1, Figure S1: Relationship between amino acid intake in week 1 and Week 1 blood spot amino acid concentrations; Figure S2. Relationship between amino acid intake in week 1 and Bayley III scores. Figure S3: Relationship between amino acid intake in week 1 and Week 1 blood spot amino acid concentrations. Table S1: Clinical and neurodevelopmental outcomes of ProVIDe trial babies recruited in New Zealand, Table S2: Blood spot amino acids concentrations of the ProVIDe cohort born in NZ, Table S3: Odds ratios of clinical outcomes by amino acid intake tertile in week 1, Table S4: Odds ratios of early-onset probable sepsis by amino acid intake tertile adjusted for baseline IV amino acid brand, Table S5: Odds ratios of late-onset probable sepsis by amino acid intake tertile adjusted for baseline IV amino acid brand, Table S6: Odds ratios of neurodisability at 2-year follow-up and individual amino acid intakes in the first week after birth, Table S7: Odds ratios of neurodisability at 2-year follow-up and week one blood spot amino acid concentrations. Table S8: Odds ratios of clinical outcome by amino acid brand in the placebo group. Refs. [11,16,18,19,20,21,22,25,26,27,28,29,30,55,56,57,58,59] are cited in Supplementary Materials file.

## Abbreviations

The following abbreviations are used in this manuscript:

BPD	bronchopulmonary dysplasia
CA	corrected age
CI	confidence interval
ELBW	extremely low birthweight
IVH	intraventricular haemorrhage
NEC	necrotising enterocolitis
NICU	neonatal intensive care unit
NZ	New Zealand
OR	odds ratio
PDA	patent ductus arteriosus
RCT	randomised controlled trial
ROP	retinopathy of prematurity
SD	standard deviation
