# Supplementary material for: Early Nutrition, Blood Amino Acids and Outcomes in Preterm Babies: Secondary Cohort Analysis of the ProVIDe RCT
Source: Nutrients. 2026 May 9;18(10):1517. doi: 10.3390/nu18101517 (PMC13210157; doi:10.3390/nu18101517)
Supplement: Supplementary file 1 [file nutrients-18-01517-s001.zip › nutrients-4205980-supplementary.pdf]

Supplementary Tables and Figures

Supplementary Table S1. Clinical and neurodevelopmental outcomes of ProVIDe trial babies recruited in New Zealand

**Clinical outcome**	**n=**	**Incidence**
Intraventricular haemorrhage ≥grade 3[a]	371	40 (11)
Bronchopulmonary dysplasia[b]	320	237 (74)
Retinopathy of prematurity ≥grade 3[c]	329	47 (15)
Necrotising enterocolitis[d]	371	44 (12)
Patent ductus arteriosus[e]	359	161 (46)
Early onset sepsis – culture proven[f]	370	10 (3)
Early onset sepsis – probable[g]	370	105 (28)
Late onset sepsis – culture proven[h]	370	125 (33)
Late onset sepsis – probable[i]	370	169 (46)
Death before discharge	382	68 (18)
**Neurodevelopmental outcome**		
No neurodisability	294	181 (62)
Neurodisability	294	113 (38)
Blindness	294	2 (0.7)
Deafness	294	7 (2)
Cerebral palsy	294	18 (6)
Bayley III Cognitive score <85	294	44 (15)
Bayley III Language score <85	294	94 (32)
Bayley III Motor score <85	294	44 (15)
Brief-P Global Executive score ≥65	294	43 (15)

Data are presented as n (%). [a]Intraventricular haemorrhage ≥grade 3 defined using the grading system from Papile et al [1]. [b]Bronchopulmonary dysplasia defined as need for oxygen at 36 weeks' corrected age or 28 28 days after birth if born after 32 weeks' gestation. [c]Retinopathy of prematurity ≥grade 3 defined using the International Classification of retinopathy of prematurity [2]. [d]Nectrotising enterocolitis defined as Bell stage 2 or higher [3]. [e]Patent ductus arteriosus diagnosed by echocardiography and with clinical decision to treat. [f]Sepsis that occurred ≤48 hours after birth to 36 weeks' and confirmed by culture (positive bacterial culture in cerebrospinal fluid, blood, or urine with clinical signs of infection and treated with antibiotics for ≥5 days. [g]Sepsis that occurred ≤48 hours after birth to 36 weeks' and believed probable. [h]Sepsis that occurred >48 hours after birth to 36 weeks' and confirmed by culture (positive bacterial culture in cerebrospinal fluid, blood, or urine with clinical signs of infection and treated with antibiotics for ≥5 days. [i]Sepsis that occurred >48 hours after birth to 36 weeks' and believed probable.

Neurodevelopmental assessment included Bayley Scales of Infant Development 3rd edition (Bayley III) [4], neurological examination, executive function (clinical assessment of inhibitory control and attentional flexibility) [5] and Behavior Rating Inventory of Executive Function—Preschool Version (BRIEF-P) [6]. Cerebral palsy and other disability outcomes were assessed according to previously reported criteria (Bloomfield et al., 2015). Neurodisabilities were classified as severe, moderate or mild [7] and confirmed by an end-point review committee whose members were unaware of trial-group assignments. Neurodisability was defined as the presence of cerebral palsy, blindness or deafness or developmental delay (Bayley III cognitive, motor or language score <85).

Supplementary Table S2 Blood spot amino acids concentrations of the ProVIDe cohort born in NZ

Amino acid (μmol.L^{-1})	Day 0-2	Week 1	Week 2-3	Week 4
Alanine	283 (211, 386)	227 (169, 322)	176 (140, 222)	176 (141, 220)
Valine	144 (119, 183)	199 (156, 297)	117 (83, 151)	100 (63, 128)
Leucine	134 (104, 184)	221 (167, 437)	148 (118, 186)	138 (104, 185)
Phenylalanine	72 (60, 88)	73 (61, 108)	49 (41, 63)	43 (34, 57)
Tyrosine	103 (78, 132)	47 (22, 85)	75 (44, 108)	75 (46, 105)
Methionine	25 (19, 36)	30 (20, 66)	20 (16, 28)	22 (16, 29)
Citrulline	19 (15, 25)	17 (12, 23)	14 (11, 18)	16 (12, 23)

Data are median (IQR).

Supplementary Table S3. Odds ratios of clinical outcomes by amino acid intake tertile in week 1

Clinical outcome	Tertile (mg.kg^{-1}.d^{-1})	Odds ratio	*p*-value
**Intraventricular haemorrhage**			
Lysine	<317.4	Reference	-
	317.4-389.6	0.98 (0.59-1.63)	0.95
	>389.6	1.24 (0.75-2.03)	0.40
Glutamic acid	<191.1	Reference	-
	191.1-278.3	1.67 (1.01-2.77)	0.05
	>278.3	1.34 (0.80-2.23)	0.27
Leucine	<307.6	Reference	-
	307.6-410.4	1.21 (0.74-1.99)	0.45
	>410.4	1.04 (0.63-1.71)	0.89
Arginine	<258.3	Reference	-
	258.3-348.1	1.21 (0.74-1.99)	0.45
	>348.1	1.04 (0.63-1.71)	0.89
Alanine	<185.7	Reference	-
	185.7-230.6	1.25 (0.75-2.06)	0.39
	>230.6	1.20 (0.72-1.98)	0.49
Valine	<214.7	Reference	-
	214.7-262.8	1.00 (0.61-1.66)	0.98
	>262.8	1.21 (0.74-1.99)	0.45
Isoleucine	<200.2	Reference	-
	200.2-253	1.13 (0.68-1.87)	0.63
	>253	1.19 (0.72-1.96)	0.49
Aspartic acid	<121	Reference	-
	121-167.9	1.34 (0.81-2.21)	0.25
	>167.9	1.15 (0.69-1.90)	0.60
Phenylalanine	<123.7	Reference	-
	123.7-152.4	1.05 (0.63-1.75)	0.84
	>152.4	1.41 (0.85-2.31)	0.18
Glycine	<106.1	Reference	-
	106.1-130.1	0.95 (0.57-1.57)	0.84
	>130.1	1.16 (0.71-1.90)	0.56
Serine	<109.6	Reference	-
	109.6-132.6	0.98 (0.59-1.62)	0.95
	>132.6	1.24 (0.75-2.03)	0.40
Histidine	<84.7	Reference	-
	84.7-108.7	1.42 (0.86-2.35)	0.17
	>108.7	1.28 (0.77-2.13)	0.34
Threonine	<108.6	Reference	-
	108.6-134	1.09 (0.66-1.81)	0.74
	>134	1.36 (0.83-2.25)	0.22
Proline	<107.9	Reference	-
	107.9-168.6	1.04 (0.63-1.72)	0.86
	>168.6	1.06 (0.64-1.73)	0.83
Methionine	<73.9	Reference	-
	73.9-98.9	1.21 (0.74-1.99)	0.45
	>98.9	1.04 (0.63-1.71)	0.89
Tryptophan	<55.9	Reference	-
	55.9-68.1	0.92 (0.56-1.52)	0.75
	>68.1	1.19 (0.73-1.96)	0.48
Cysteine	<8.6	Reference	-
	8.6-50.9	1.19 (0.72-1.94)	0.50
	>50.9	0.93 (0.56-1.53)	0.77
Taurine	<11.1	Reference	-
	11.1-16.7	1.71 (1.04-2.83)	0.04
	>16.7	1.09 (0.65-1.84)	0.74
Tyrosine	<21	Reference	-
	21-49.3	1.12 (0.68-1.85)	0.64
	>49.3	1.01 (0.61-1.67)	0.96
**Bronchopulmonary dysplasia**			

Lysine	<317.4	Reference	-
	317.4-389.6	1.24 (0.71-2.16)	0.45
	>389.6	1.59 (0.89-2.82)	0.11
Glutamic acid	<191.1	Reference	-
	191.1-278.3	1.11 (0.63-1.98)	0.72
	>278.3	0.91 (0.52-1.58)	0.73
Leucine	<307.6	Reference	-
	307.6-410.4	1.08 (0.62-1.88)	0.79
	>410.4	1.56 (0.88-2.76)	0.13
Arginine	<258.3	Reference	-
	258.3-348.1	1.08 (0.62-1.88)	0.79
	>348.1	1.56 (0.88-2.76)	0.13
Alanine	<185.7	Reference	-
	185.7-230.6	0.76 (0.43-1.36)	0.36
	>230.6	0.74 (0.41-1.31)	0.29
Valine	<214.7	Reference	-
	214.7-262.8	1.32 (0.76-2.30)	0.33
	>262.8	1.49 (0.84-2.63)	0.17
Isoleucine	<200.2	Reference	-
	200.2-253	1.02 (0.58-1.77)	0.95
	>253	1.53 (0.86-2.72)	0.14
Aspartic acid	<121	Reference	-
	121-167.9	1.10 (0.62-1.96)	0.75
	>167.9	0.89 (0.51-1.55)	0.67
Phenylalanine	<123.7	Reference	-
	123.7-152.4	1.33 (0.76-2.34)	0.32
	>152.4	1.49 (0.85-2.61)	0.17
Glycine	<106.1	Reference	-
	106.1-130.1	1.13 (0.65-1.97)	0.67
	>130.1	1.25 (0.70-2.21)	0.46
Serine	<109.6	Reference	-
	109.6-132.6	1.41 (0.80-2.47)	0.23
	>132.6	1.44 (0.81-2.54)	0.21
Histidine	<84.7	Reference	-
	84.7-108.7	0.91 (0.51-1.62)	0.75
	>108.7	0.81 (0.46-1.42)	0.45
Threonine	<108.6	Reference	-
	108.6-134	1.33 (0.76-2.34)	0.32
	>134	1.49 (0.85-2.61)	0.17
Proline	<107.9	Reference	-
	107.9-168.6	1.45 (0.84-2.52)	0.18
	>168.6	2.25 (1.26-4.03)	0.006
Methionine	<73.9	Reference	-
	73.9-98.9	1.08 (0.62-1.88)	0.79
	>98.9	1.56 (0.88-2.76)	0.13
Tryptophan	<55.9	Reference	-
	55.9-68.1	1.43 (0.82-2.50)	0.21
	>68.1	1.55 (0.87-2.73)	0.13
Cysteine	<8.6	Reference	-
	8.6-50.9	0.68 (0.37-1.25)	0.22
	>50.9	0.47 (0.26-0.82)	0.009
Taurine	<11.1	Reference	-
	11.1-16.7	0.95 (0.54-1.67)	0.85
	>16.7	0.97 (0.55-1.73)	0.92
Tyrosine	<21	Reference	-
	21-49.3	1.59 (0.91-2.76)	0.10
	>49.3	2.21 (1.24-3.93)	0.007
**Retinopathy of prematurity**			
Lysine	<317.4	Reference	-
	317.4-389.6	0.66 (0.34-1.26)	0.21
	>389.6	0.53 (0.27-1.06)	0.07
Glutamic acid	<191.1	Reference	-
	191.1-278.3	1.66 (0.86-3.20)	0.13
	>278.3	0.77 (0.37-1.62)	0.50

Leucine	<307.6	Reference	-
	307.6-410.4	0.65 (0.34-1.27)	0.21
	>410.4	0.57 (0.29-1.12)	0.10
Arginine	<258.3	Reference	-
	258.3-348.1	0.65 (0.34-1.27)	0.21
	>348.1	0.57 (0.29-1.12)	0.10
Alanine	<185.7	Reference	-
	185.7-230.6	1.41 (0.73-2.71)	0.30
	>230.6	0.72 (0.35-1.49)	0.38
Valine	<214.7	Reference	-
	214.7-262.8	0.60 (0.31-1.18)	0.14
	>262.8	0.58 (0.30-1.14)	0.11
Isoleucine	<200.2	Reference	-
	200.2-253	0.67 (0.34-1.31)	0.24
	>253	0.57 (0.29-1.12)	0.11
Aspartic acid	<121	Reference	-
	121-167.9	1.55 (0.81-2.97)	0.18
	>167.9	0.69 (0.33-1.45)	0.33
Phenylalanine	<123.7	Reference	-
	123.7-152.4	0.59 (0.30-1.15)	0.12
	>152.4	0.56 (0.28-1.08)	0.09
Glycine	<106.1	Reference	-
	106.1-130.1	0.86 (0.45-1.64)	0.64
	>130.1	0.63 (0.31-1.26)	0.19
Serine	<109.6	Reference	-
	109.6-132.6	0.77 (0.40-1.48)	0.44
	>132.6	0.59 (0.29-1.18)	0.14
Histidine	<84.7	Reference	-
	84.7-108.7	1.26 (0.66-2.42)	0.48
	>108.7	0.68 (0.33-1.39)	0.29
Threonine	<108.6	Reference	-
	108.6-134	0.63 (0.32-1.21)	0.17
	>134	0.52 (0.26-1.02)	0.06
Proline	<107.9	Reference	-
	107.9-168.6	0.73 (0.38-1.42)	0.36
	>168.6	0.64 (0.32-1.26)	0.19
Methionine	<73.9	Reference	-
	73.9-98.9	0.65 (0.34-1.27)	0.21
	>98.9	0.57 (0.29-1.12)	0.10
Tryptophan	<55.9	Reference	-
	55.9-68.1	0.59 (0.30-1.15)	0.12
	>68.1	0.57 (0.29-1.13)	0.11
Cysteine	<8.6	Reference	-
	8.6-50.9	1.85 (0.94-3.61)	0.07
	>50.9	0.98 (0.48-2.02)	0.96
Taurine	<11.1	Reference	-
	11.1-16.7	1.58 (0.82-3.07)	0.17
	>16.7	0.81 (0.38-1.71)	0.58
Tyrosine	<21	Reference	-
	21-49.3	0.77 (0.40-1.49)	0.44
	>49.3	0.64 (0.33-1.27)	0.20
**Necrotising enterocolitis**			
Lysine	<317.4	Reference	-
	317.4-389.6	0.99 (0.50-1.99)	0.99
	>389.6	0.88 (0.44-1.80)	0.73
Glutamic acid	<191.1	Reference	-
	191.1-278.3	1.22 (0.60-2.47)	0.58
	>278.3	1.07 (0.52-2.20)	0.85
Leucine	<307.6	Reference	-
	307.6-410.4	1.22 (0.61-2.42)	0.58
	>410.4	0.89 (0.43-1.84)	0.75
Arginine	<258.3	Reference	-
	258.3-348.1	1.22 (0.61-2.42)	0.58
	>348.1	0.89 (0.43-1.84)	0.75

Alanine	<185.7	Reference	-
	185.7-230.6	1.31 (0.64-2.68)	0.46
	>230.6	1.23 (0.60-2.52)	0.58
Valine	<214.7	Reference	-
	214.7-262.8	0.93 (0.47-1.84)	0.83
	>262.8	0.78 (0.38-1.59)	0.50
Isoleucine	<200.2	Reference	-
	200.2-253	1.07 (0.54-2.11)	0.86
	>253	0.82 (0.40-1.68)	0.59
Aspartic acid	<121	Reference	-
	121-167.9	1.60 (0.78-3.27)	0.20
	>167.9	1.27 (0.61-2.66)	0.53
Phenylalanine	<123.7	Reference	-
	123.7-152.4	1.06 (0.53-2.10)	0.87
	>152.4	0.83 (0.40-1.69)	0.60
Glycine	<106.1	Reference	-
	106.1-130.1	1.06 (0.53-2.14)	0.86
	>130.1	1.01 (0.50-2.05)	0.98
Serine	<109.6	Reference	-
	109.6-132.6	1.06 (0.53-2.10)	0.87
	>132.6	0.83 (0.40-1.69)	0.60
Histidine	<84.7	Reference	-
	84.7-108.7	1.50 (0.73-3.09)	0.27
	>108.7	1.32 (0.64-2.76)	0.45
Threonine	<108.6	Reference	-
	108.6-134	1.06 (0.53-2.10)	0.87
	>134	0.83 (0.40-1.69)	0.60
Proline	<107.9	Reference	-
	107.9-168.6	0.98 (0.50-1.95)	0.96
	>168.6	0.80 (0.39-1.62)	0.53
Methionine	<73.9	Reference	-
	73.9-98.9	1.22 (0.61-2.42)	0.58
	>98.9	0.89 (0.43-1.84)	0.75
Tryptophan	<55.9	Reference	-
	55.9-68.1	1.06 (0.53-2.10)	0.87
	>68.1	0.83 (0.40-1.69)	0.60
Cysteine	<8.6	Reference	-
	8.6-50.9	2.22 (1.11-4.45)	0.02
	>50.9	0.91 (0.41-2.02)	0.82
Taurine	<11.1	Reference	-
	11.1-16.7	1.27 (0.62-2.58)	0.51
	>16.7	1.11 (0.54-2.31)	0.77
Tyrosine	<21	Reference	-
	21-49.3	0.99 (0.50-1.97)	0.98
	>49.3	0.81 (0.40-1.65)	0.56
**Patent ductus arteriosus**			
Lysine	<317.4	Reference	-
	317.4-389.6	0.78 (0.49-1.27)	0.32
	>389.6	1.01 (0.63-1.64)	0.95
Glutamic acid	<191.1	Reference	-
	191.1-278.3	2.09 (1.29-3.40)	0.003
	>278.3	1.42 (0.87-2.30)	0.16
Leucine	<307.6	Reference	-
	307.6-410.4	0.85 (0.53-1.37)	0.85
	>410.4	0.78 (0.48-1.26)	0.31
Arginine	<258.3	Reference	-
	258.3-348.1	0.85 (0.53-1.37)	0.85
	>348.1	0.78 (0.48-1.26)	0.31
Alanine	<185.7	Reference	-
	185.7-230.6	1.46 (0.90-2.36)	0.12
	>230.6	1.28 (0.79-2.07)	0.31
Valine	<214.7	Reference	-
	214.7-262.8	0.86 (0.53-1.38)	0.53
	>262.8	1.02 (0.63-1.64)	0.95

Isoleucine	<200.2	Reference	-
	200.2-253	0.84 (0.52-1.36)	0.48
	>253	0.90 (0.56-1.46)	0.68
Aspartic acid	<121	Reference	-
	121-167.9	1.94 (1.19-3.14)	0.007
	>167.9	1.45 (0.90-2.35)	0.13
Phenylalanine	<123.7	Reference	-
	123.7-152.4	0.93 (0.58-1.50)	0.78
	>152.4	0.97 (0.60-1.57)	0.91
Glycine	<106.1	Reference	-
	106.1-130.1	0.75 (0.47-1.21)	0.24
	>130.1	0.97 (0.60-1.57)	0.90
Serine	<109.6	Reference	-
	109.6-132.6	0.78 (0.48-1.25)	0.30
	>132.6	1.03 (0.64-1.66)	0.91
Histidine	<84.7	Reference	-
	84.7-108.7	1.65 (1.02-2.66)	0.04
	>108.7	1.37 (0.84-2.21)	0.20
Threonine	<108.6	Reference	-
	108.6-134	0.93 (0.58-1.50)	0.78
	>134	0.97 (0.60-1.57)	0.91
Proline	<107.9	Reference	-
	107.9-168.6	0.89 (0.55-1.43)	0.63
	>168.6	0.68 (0.42-1.10)	0.12
Methionine	<73.9	Reference	-
	73.9-98.9	0.85 (0.53-1.37)	0.51
	>98.9	0.78 (0.48-1.26)	0.31
Tryptophan	<55.9	Reference	-
	55.9-68.1	0.90 (0.56-1.45)	0.66
	>68.1	1.06 (0.66-1.72)	0.80
Cysteine	<8.6	Reference	-
	8.6-50.9	2.02 (1.24-3.28)	0.005
	>50.9	1.63 (1.01-2.62)	0.05
Taurine	<11.1	Reference	-
	11.1-16.7	2.52 (1.55-4.12)	0.0002
	>16.7	1.52 (0.93-2.50)	0.09
Tyrosine	<21	Reference	-
	21-49.3	1.06 (0.66-1.71)	0.80
	>49.3	0.65 (0.41-1.05)	0.08
**Early onset sepsis - culture proven**			
Lysine	<317.4	Reference	-
	317.4-389.6	0.30 (0.06-1.53)	0.15
	>389.6	0.78 (0.23-2.61)	0.68
Glutamic acid	<191.1	Reference	-
	191.1-278.3	2.41 (0.46-12.63)	0.30
	>278.3	2.89 (0.57-14.58)	0.20
Leucine	<307.6	Reference	-
	307.6-410.4	0.62 (0.17-2.26)	0.47
	>410.4	0.46 (0.11-1.89)	0.28
Arginine	<258.3	Reference	-
	258.3-348.1	0.62 (0.17-2.26)	0.47
	>348.1	0.46 (0.11-1.89)	0.28
Alanine	<185.7	Reference	-
	185.7-230.6	2.91 (0.58-14.69)	0.20
	>230.6	2.39 (0.46-12.54)	0.30
Valine	<214.7	Reference	-
	214.7-262.8	0.93 (0.23-3.79)	0.92
	>262.8	1.20 (0.31-4.55)	0.79
Isoleucine	<200.2	Reference	-
	200.2-253	0.30 (0.06-1.54)	0.15
	>253	0.77 (0.23-2.60)	0.68
Aspartic acid	<121	Reference	-
	121-167.9	2.43 (0.46-12.73)	0.29
	>167.9	2.93 (0.58-14.80)	0.19

Phenylalanine	<123.7	Reference	-
	123.7-152.4	0.30 (0.06-1.53)	0.15
	>152.4	0.78 (0.23-2.61)	0.69
Glycine	<106.1	Reference	-
	106.1-130.1	1.18 (0.31-4.48)	0.81
	>130.1	0.94 (0.23-3.85)	0.93
Serine	<109.6	Reference	-
	109.6-132.6	0.94 (0.23-3.82)	0.93
	>132.6	1.19 (0.31-4.52)	0.80
Histidine	<84.7	Reference	-
	84.7-108.7	2.91 (0.58-14.69)	0.20
	>108.7	2.39 (0.46-12.54)	0.30
Threonine	<108.6	Reference	-
	108.6-134	0.30 (0.06-1.53)	0.15
	>134	0.78 (0.23-2.61)	0.69
Proline	<107.9	Reference	-
	107.9-168.6	0.82 (0.24-2.76)	0.75
	>168.6	0.31 (0.06-1.58)	0.16
Methionine	<73.9	Reference	-
	73.9-98.9	0.62 (0.17-2.26)	0.47
	>98.9	0.46 (0.11-1.89)	0.28
Tryptophan	<55.9	Reference	-
	55.9-68.1	0.94 (0.23-3.82)	0.93
	>68.1	1.19 (0.31-4.52)	0.80
Cysteine	<8.6	Reference	-
	8.6-50.9	1.73 (0.41-7.38)	0.46
	>50.9	1.67 (0.39-7.11)	0.49
Taurine	<11.1	Reference	-
	11.1-16.7	1.17 (0.26-5.32)	0.84
	>16.7	1.85 (0.45-7.57)	0.39
Tyrosine	<21	Reference	-
	21-49.3	0.82 (0.24-2.76)	0.75
	>49.3	0.32 (0.06-1.62)	0.17
**Early onset sepsis - probable**			
Lysine	<317.4	Reference	-
	317.4-389.6	1.31 (0.73-2.34)	0.37
	>389.6	2.67 (1.54-4.63)	0.0005
Glutamic acid	<191.1	Reference	-
	191.1-278.3	0.29 (0.17-0.50)	<0.0001
	>278.3	0.16 (0.09-0.29)	<0.0001
Leucine	<307.6	Reference	-
	307.6-410.4	1.16 (0.62-2.19)	0.65
	>410.4	4.69 (2.66-8.30)	<0.0001
Arginine	<258.3	Reference	-
	258.3-348.1	1.16 (0.62-2.19)	0.65
	>348.1	4.69 (2.66-8.30)	<0.0001
Alanine	<185.7	Reference	-
	185.7-230.6	0.54 (0.33-0.90)	0.02
	>230.6	0.24 (0.13-0.43)	<0.0001
Valine	<214.7	Reference	-
	214.7-262.8	1.22 (0.69-2.14)	0.50
	>262.8	2.01 (1.17-3.46)	0.011
Isoleucine	<200.2	Reference	-
	200.2-253	1.24 (0.68-2.27)	0.49
	>253	3.48 (1.99-6.08)	<0.0001
Aspartic acid	<121	Reference	-
	121-167.9	0.28 (0.16-0.47	<0.0001
	>167.9	0.16 (0.09-0.29)	<0.0001
Phenylalanine	<123.7	Reference	-
	123.7-152.4	1.32 (0.73-2.39)	0.36
	>152.4	2.98 (1.71-5.18)	0.0001
Glycine	<106.1	Reference	-
	106.1-130.1	0.88 (0.51-1.52)	0.65
	>130.1	1.23 (0.73-2.08)	0.44

Serine	<109.6	Reference	-
	109.6-132.6	1.07 (0.62-1.85)	0.80
	>132.6	1.43 (0.84-2.44)	0.19
Histidine	<84.7	Reference	-
	84.7-108.7	0.45 (0.27-0.74)	0.002
	>108.7	0.19 (0.10-0.35)	<0.0001
Threonine	<108.6	Reference	-
	108.6-134	1.32 (0.73-2.39)	0.36
	>134	2.98 (1.71-5.18)	0.0001
Proline	<107.9	Reference	-
	107.9-168.6	2.53 (1.25-5.11)	0.009
	>168.6	9.29 (4.81-17.95)	<0.0001
Methionine	<73.9	Reference	-
	73.9-98.9	1.16 (0.62-2.19)	0.65
	>98.9	4.69 (2.66-8.30)	<0.0001
Tryptophan	<55.9	Reference	-
	55.9-68.1	1.17 (0.67-2.05)	0.57
	>68.1	1.85 (1.08-3.16)	0.03
Cysteine	<8.6	Reference	-
	8.6-50.9	0.13 (0.07-0.24)	<0.0001
	>50.9	0.08 (0.04-0.15)	<0.0001
Taurine	<11.1	Reference	-
	11.1-16.7	0.50 (0.30-0.83)	0.007
	>16.7	0.15 (0.08-0.29)	<0.0001
Tyrosine	<21	Reference	-
	21-49.3	2.59 (1.56-4.31)	0.0002
	>49.3	4.59 (2.75-7.67)	<0.0001
**Late onset sepsis – culture proven**			
Lysine	<317.4	Reference	-
	317.4-389.6	1.26 (0.76-2.09)	0.36
	>389.6	1.36 (0.82-2.24)	0.23
Glutamic acid	<191.1	Reference	-
	191.1-278.3	1.26 (0.77-2.07)	0.36
	>278.3	1.00 (0.61-1.66)	0.99
Leucine	<307.6	Reference	-
	307.6-410.4	1.47 (0.89-2.45)	0.13
	>410.4	1.47 (0.89-2.45)	0.13
Arginine	<258.3	Reference	-
	258.3-348.1	1.47 (0.89-2.45)	0.13
	>348.1	1.47 (0.89-2.45)	0.13
Alanine	<185.7	Reference	-
	185.7-230.6	1.30 (0.79-2.13)	0.30
	>230.6	0.97 (0.59-1.61)	0.91
Valine	<214.7	Reference	-
	214.7-262.8	1.25 (0.76-2.07)	0.39
	>262.8	1.37 (0.83-2.27)	0.22
Isoleucine	<200.2	Reference	-
	200.2-253	1.45 (0.88-2.40)	0.15
	>253	1.31 (0.79-2.17)	0.30
Aspartic acid	<121	Reference	-
	121-167.9	1.35 (0.82-2.22)	0.23
	>167.9	0.96 (0.58-1.60)	0.88
Phenylalanine	<123.7	Reference	-
	123.7-152.4	1.39 (0.84-2.30)	0.20
	>152.4	1.36 (0.82-2.26)	0.23
Glycine	<106.1	Reference	-
	106.1-130.1	1.54 (0.93-2.56)	0.09
	>130.1	1.51 (0.91-2.52)	0.11
Serine	<109.6	Reference	-
	109.6-132.6	1.35 (0.81-2.24)	0.24
	>132.6	1.41 (0.85-2.33)	0.19
Histidine	<84.7	Reference	-
	84.7-108.7	1.48 (0.90-2.43)	0.13
	>108.7	1.04 (0.63-1.73)	0.87

Threonine	<108.6	Reference	-
	108.6-134	1.39 (0.84-2.30)	0.20
	>134	1.36 (0.82-2.26)	0.23
Proline	<107.9	Reference	-
	107.9-168.6	1.39 (0.83-2.33)	0.21
	>168.6	1.88 (1.34-3.11)	0.014
Methionine	<73.9	Reference	-
	73.9-98.9	1.47 (0.89-2.45)	0.13
	>98.9	1.47 (0.89-2.45)	0.13
Tryptophan	<55.9	Reference	-
	55.9-68.1	1.35 (0.81-2.24)	0.24
	>68.1	1.41 (0.85-2.33)	0.19
Cysteine	<8.6	Reference	-
	8.6-50.9	0.97 (0.60-1.59)	0.91
	>50.9	0.73 (0.45-1.21)	0.22
Taurine	<11.1	Reference	-
	11.1-16.7	1.54 (0.93-2.55)	0.09
	>16.7	1.15 (0.69-1.92)	0.60
Tyrosine	<21	Reference	-
	21-49.3	1.53 (0.92-2.55)	0.10
	>49.3	1.78 (1.07-2.95)	0.03
**Late onset sepsis – probable**			
Lysine	<317.4	Reference	-
	317.4-389.6	1.52 (0.93-2.48)	0.09
	>389.6	2.41 (1.48-3.93)	0.0004
Glutamic acid	<191.1	Reference	-
	191.1-278.3	0.36 (0.22-0.58)	<0.0001
	>278.3	0.44 (0.27-0.71)	0.0007
Leucine	<307.6	Reference	-
	307.6-410.4	1.97 (1.20-3.25)	0.008
	>410.4	3.47 (2.10-5.72)	<0.0001
Arginine	<258.3	Reference	-
	258.3-348.1	1.97 (1.20-3.25)	0.008
	>348.1	3.47 (2.10-5.72)	<0.0001
Alanine	<185.7	Reference	-
	185.7-230.6	0.56 (0.35-0.91)	0.02
	>230.6	0.63 (0.39-1.01)	0.05
Valine	<214.7	Reference	-
	214.7-262.8	1.24 (0.77-2.02)	0.38
	>262.8	2.03 (1.25-3.29)	0.004
Isoleucine	<200.2	Reference	-
	200.2-253	1.86 (1.13-3.04)	0.012
	>253	2.63 (1.61-4.31)	0.0001
Aspartic acid	<121	Reference	-
	121-167.9	0.40 (0.25-0.65)	0.0002
	>167.9	0.49 (0.31-0.79)	0.004
Phenylalanine	<123.7	Reference	-
	123.7-152.4	1.66 (1.02-2.71)	0.04
	>152.4	2.43 (1.49-3.96)	0.0004
Glycine	<106.1	Reference	-
	106.1-130.1	0.85 (0.52-1.37)	0.50
	>130.1	1.56 (0.97-2.51)	0.07
Serine	<109.6	Reference	-
	109.6-132.6	1.08 (0.67-1.75)	0.76
	>132.6	1.77 (1.10-2.85)	0.02
Histidine	<84.7	Reference	-
	84.7-108.7	0.60 (0.37-0.96)	0.03
	>108.7	0.59 (0.37-0.95)	0.03
Threonine	<108.6	Reference	-
	108.6-134	0.62 (0.99-2.64)	0.05
	>134	2.50 (1.53-4.07)	0.0002
Proline	<107.9	Reference	-
	107.9-168.6	2.75 (1.65-4.58)	<0.0001
	>168.6	4.15 (2.49-6.91)	<0.0001
Methionine	<73.9	Reference	-

	73.9-98.9	1.97 (1.20-3.25)	0.008
	>98.9	3.47 (2.10-5.72)	<0.0001
Tryptophan	<55.9	Reference	-
	55.9-68.1	1.22 (0.75-1.99)	0.41
	>68.1	2.06 (1.27-3.33)	0.003
Cysteine	<8.6	Reference	-
	8.6-50.9	0.32 (0.20-0.52)	<0.0001
	>50.9	0.34 (0.21-0.56)	<0.0001
Taurine	<11.1	Reference	-
	11.1-16.7	0.49 (0.30-0.78)	0.003
	>16.7	0.49 (0.30-0.79)	0.004
Tyrosine	<21	Reference	-
	21-49.3	2.59 (1.56-4.31)	0.0002
	>49.3	4.59 (2.75-7.67)	<0.0001
**Death before discharge**			
Lysine	<317.4	Reference	-
	317.4-389.6	0.68 (0.37-1.26)	0.22
	>389.6	0.67 (0.37-1.23)	0.20
Glutamic acid	<191.1	Reference	-
	191.1-278.3	1.09 (0.60-1.99)	0.78
	>278.3	0.93 (0.50-1.73)	0.82
Leucine	<307.6	Reference	-
	307.6-410.4	0.78 (0.43-1.40)	0.40
	>410.4	0.57 (0.30-1.06)	0.08
Arginine	<258.3	Reference	-
	258.3-348.1	0.78 (0.43-1.40)	0.40
	>348.1	0.57 (0.30-1.06)	0.08
Alanine	<185.7	Reference	-
	185.7-230.6	0.87 (0.47-1.61)	0.66
	>230.6	0.90 (0.49-1.65)	0.73
Valine	<214.7	Reference	-
	214.7-262.8	0.65 (0.35-1.20)	0.16
	>262.8	0.71 (0.39-1.29)	0.26
Isoleucine	<200.2	Reference	-
	200.2-253	0.96 (0.53-1.72)	0.88
	>253	0.62 (0.33-1.16)	0.13
Aspartic acid	<121	Reference	-
	121-167.9	1.22 (0.67-2.23)	0.52
	>167.9	0.98 (0.52-1.85)	0.96
Phenylalanine	<123.7	Reference	-
	123.7-152.4	0.90 (0.50-1.64)	0.74
	>152.4	0.66 (0.35-1.23)	0.19
Glycine	<106.1	Reference	-
	106.1-130.1	0.51 (0.27-0.97)	0.09
	>130.1	0.80 (0.45-1.43)	0.45
Serine	<109.6	Reference	-
	109.6-132.6	0.58 (0.31-1.09)	0.09
	>132.6	0.72 (0.40-1.29)	0.27
Histidine	<84.7	Reference	-
	84.7-108.7	0.97 (0.53-1.78)	0.92
	>108.7	0.93 (0.50-1.72)	0.82
Threonine	<108.6	Reference	-
	108.6-134	0.90 (0.50-1.64)	0.74
	>134	0.66 (0.35-1.23)	0.19
Proline	<107.9	Reference	-
	107.9-168.6	0.95 (0.52-1.72)	0.86
	>168.6	0.78 (0.42-1.45)	0.43
Methionine	<73.9	Reference	-
	73.9-98.9	0.78 (0.43-1.40)	0.40
	>98.9	0.57 (0.30-1.06)	0.08
Tryptophan	<55.9	Reference	-
	55.9-68.1	0.62 (0.33-1.15)	0.13
	>68.1	0.74 (0.41-1.34)	0.32
Cysteine	<8.6	Reference	-
	8.6-50.9	1.59 (0.88-2.87)	0.12

	>50.9	0.76 (0.39-1.47)	0.41
Taurine	<11.1	Reference	-
	11.1-16.7	1.09 (0.60-2.01)	0.77
	>16.7	0.96 (0.52-1.80)	0.90
Tyrosine	<21	Reference	-
	21-49.3	1.09 (0.60-1.97)	0.78
	>49.3	0.77 (0.41-1.45)	0.42

Data are presented as odds ratios (95% CI). Significant p-value <0.01.

Supplementary Table S4. Odds ratios of early-onset probable sepsis by amino acid intake tertile adjusted for baseline IV amino acid brand

IV Amino acid	Tertile ($mg.kg^{-1}.d^{-1}$)	Odds ratio	*p*-value
Lysine	<317.4	Reference	-
	317.4-389.6	1.16 (0.61-0.92)	0.66
	>389.6	2.25 (1.19-4.25)	0.01
Glutamic acid	<191.1	Reference	-
	191.1-278.3	0.51 (0.29-0.92)	0.02
	>278.3	0.41 (0.19-0.88)	0.02
Leucine	<307.6	Reference	-
	307.6-410.4	1.23 (0.59-2.55)	0.58
	>410.4	2.75 (1.36-5.57)	0.005
Arginine	<258.3	Reference	-
	258.3-348.1	1.19 (0.57-2.47)	0.65
	>348.1	2.86 (1.40-5.84)	0.004
Alanine	<185.7	Reference	-
	185.7-230.6	0.71 (0.41-1.22)	0.22
	>230.6	0.59 (0.29-1.21)	0.15
Valine	<214.7	Reference	-
	214.7-262.8	1.11 (0.60-2.07)	0.73
	>262.8	1.92 (1.03-3.60)	0.04
Isoleucine	<200.2	Reference	-
	200.2-253	1.19 (0.60-2.36)	0.61
	>253	2.25 (1.15-4.40)	0.02
Aspartic acid	<121	Reference	-
	121-167.9	0.43 (0.25-0.77)	0.004
	>167.9	0.37 (0.18-0.79)	0.01
Phenylalanine	<123.7	Reference	-
	123.7-152.4	1.21 (0.63-2.33)	0.58
	>152.4	2.21 (1.16-4.22)	0.02
Glycine	<106.1	Reference	-
	106.1-130.1	0.91 (0.50-1.64)	0.76
	>130.1	1.51 (0.83-2.77)	0.18
Serine	<109.6	Reference	-
	109.6-132.6	1.07 (0.59-1.94)	0.83
	>132.6	1.56 (0.85-2.88)	0.15
Histidine	<84.7	Reference	-
	84.7-108.7	0.62 (0.36-1.06)	0.08
	>108.7	0.45 (0.21-0.95)	0.04
Threonine	<108.6	Reference	-
	108.6-134	1.19 (0.62-2.30)	0.60
	>134	2.25 (1.18-4.29)	0.01
Proline	<107.9	Reference	-
	107.9-168.6	5.52 (2.03-14.99)	0.0008
	>168.6	7.92 (2.79-22.48)	0.0001
Methionine	<73.9	Reference	-
	73.9-98.9	1.23 (0.59-2.55)	0.58
	>98.9	2.75 (1.36-5.57)	0.005
Tryptophan	<55.9	Reference	-
	55.9-68.1	1.08 (0.59-2.01)	0.80
	>68.1	1.75 (0.94-3.25)	0.08
Cysteine	<8.6	Reference	-
	8.6-50.9	0.15 (0.05-0.46)	0.0009
	>50.9	0.08 (0.03-0.23)	<0.0001
Taurine	<11.1	Reference	-
	11.1-16.7	0.77 (0.45-1.33)	0.35
	>16.7	0.38 (0.18-0.81)	0.01
Tyrosine	<21	Reference	-
	21-49.3	1.95 (0.95-4.02)	0.07
	>49.3	6.55 (2.69-15.96)	<0.0001

Data are presented as odds ratio (95%CI) Significant p value <0.05 shown in red.

Supplementary Table S5. Odds ratios of late-onset probable sepsis by amino acid intake tertile adjusted for baseline IV amino acid brand

IV Amino acid	Tertile (mg.kg^{-1}.d^{-1})	Odds ratio	*p*-value
Lysine	<317.4	Reference	-
	317.4-389.6	1.26 (0.74-2.14)	0.40
	>389.6	1.82 (1.05-3.17)	0.03
Glutamic acid	<191.1	Reference	-
	191.1-278.3	0.46 (0.26-0.79)	0.005
	>278.3	0.57 (0.30-1.08)	0.08
Leucine	<307.6	Reference	-
	307.6-410.4	1.79 (1.00-3.22)	0.05
	>410.4	2.47 (1.33-4.57)	0.004
Arginine	<258.3	Reference	-
	258.3-348.1	1.81 (1.01-3.24)	0.05
	>348.1	2.46 (1.32-4.58)	0.004
Alanine	<185.7	Reference	-
	185.7-230.6	0.64 (0.39-1.05)	0.08
	>230.6	0.85 (0.47-1.54)	0.59
Valine	<214.7	Reference	-
	214.7-262.8	1.04 (0.62-1.75)	0.89
	>262.8	1.59 (0.92-2.75)	0.10
Isoleucine	<200.2	Reference	-
	200.2-253	1.58 (0.91-2.75)	0.10
	>253	1.79 (1.00-3.21)	0.05
Aspartic acid	<121	Reference	-
	121-167.9	0.50 (0.30-0.85)	0.01
	>167.9	0.66 (0.35-1.23)	0.19
Phenylalanine	<123.7	Reference	-
	123.7-152.4	1.38 (0.80-2.36)	0.25
	>152.4	1.74 (0.99-3.04)	0.05
Glycine	<106.1	Reference	-
	106.1-130.1	0.77 (0.46-1.29)	0.32
	>130.1	1.41 (0.83-2.40)	0.21
Serine	<109.6	Reference	-
	109.6-132.6	0.95 (0.57-1.59)	0.85
	>132.6	1.51 (0.88-2.59)	0.13
Histidine	<84.7	Reference	-
	84.7-108.7	0.71 (0.43-1.18)	0.18
	>108.7	0.81 (0.44-1.49)	0.50
Threonine	<108.6	Reference	-
	108.6-134	1.34 (0.78-2.29)	0.29
	>134	1.80 (1.03-3.16)	0.04
Proline	<107.9	Reference	-
	107.9-168.6	1.24 (0.58-2.64)	0.57
	>168.6	1.77 (0.80-3.92)	0.16
Methionine	<73.9	Reference	-
	73.9-98.9	1.79 (1.00-3.22)	0.05
	>98.9	2.47 (1.33-4.57)	0.004
Tryptophan	<55.9	Reference	-
	55.9-68.1	1.03 (0.61-1.74)	0.90
	>68.1	1.63 (0.95-2.82)	0.08
Cysteine	<8.6	Reference	-
	8.6-50.9	0.30 (0.13-0.68)	0.004
	>50.9	0.33 (0.16-0.67)	0.002
Taurine	<11.1	Reference	-
	11.1-16.7	0.59 (0.36-0.99)	0.046
	>16.7	0.69 (0.38-1.26)	0.23
Tyrosine	<21	Reference	-
	21-49.3	2.56 (1.54-4.26)	0.0003
	>49.3	6.06 (1.94-8.51)	0.0002

Data are presented as odds ratio (95%CI). Significant p-value <0.01 shown in red.

Supplementary Table S6. Odds ratios of neurodisability at 2-year follow-up and individual amino acid intakes in the first week after birth

**Amino acid intake (mg.Kg.$^{-1}$.day^{-1})**	**OR (95% CI)**	***p*-value**	**Adjusted* OR (95% CI)**	***p*-value**
Tyrosine				
Quartile 1 (<16)	1.65 (0.85, 3.19)	0.14	1.63 (0.83, 3.21)	0.16
Quartile 2 (16-35)	1.41 (0.72, 2.76)	0.32	1.36 (0.68, 2.72)	0.38
Quartile 3 (36-72)	1.11 (0.56, 2.19)	0.77	1.21 (0.60, 2.45)	0.59
Quartile 4 (>72)^	-	-	-	-
Methionine + Cysteine				
Quartile 1 (<105)^	-	-	-	-
Quartile 2 (98-124)	1.30 (0.67, 2.52)	0.44	1.23 (0.62, 2.42)	0.55
Quartile 3 (125-142)	1.13 (0.57, 2.27)	0.72	1.09 (0.53, 2.24)	0.81
Quartile 4 (>142)	1.24 (0.62, 2.44)	0.54	1.37 (0.68, 2.77)	0.38
Threonine				
Quartile 1 (<98)^	-	-	-	-
Quartile 2 (98-124)	0.82 (0.42, 1.60)	0.57	0.78 (0.39, 1.55)	0.47
Quartile 3 (125-142)	0.96 (0.50, 1.85)	0.90	0.96 (0.49, 1.89)	0.90
Quartile 4 (>142)	0.69 (0.35, 1.37)	0.29	0.70 (0.35, 1.43)	0.33
Lysine				
Quartile 1 (<290)^	-	-	-	-
Quartile 2 (290-363)	0.74 (0.38, 1.45)	0.39	0.68 (0.34, 1.36)	0.27
Quartile 3 (364-413)	0.99 (0.51, 1.93)	0.99	0.98 (0.49, 1.95)	0.96
Quartile 4 (>414)	0.75 (0.38, 1.47)	0.40	0.76 (0.38, 1.52)	0.43

OR, Odds ratio; CI, Confidence interval.

*Adjusted for sex and SGA. ^Denotes reference quartile. ^Denotes reference group.

Supplementary Table S7 Odds ratios of neurodisability at 2-year follow-up and week one blood spot amino acid concentrations

Amino acid (µmol.L^{-1})	Unadjusted OR (95% CI)	*p*-value	Adjusted* OR (95% CI)	*p*-value
Alanine				
Quartile 1 (<171)	1.02 (0.38, 2.77)	0.97	1.07 (0.39, 2.96)	0.89
Quartile 2 (171-230)^	-	-	-	-
Quartile 3 (231-325)	1.41 (0.52, 3.83)	0.50	1.62 (0.58, 4.51)	0.35
Quartile 4 (>325)	1.14 (0.41, 3.14)	0.80	1.18 (0.42, 3.31)	0.76
Valine				
Quartile 1 (<156)	1.48 (0.54, 4.07)	0.44	1.33 (0.48, 3.73)	0.58
Quartile 2 (156-199)^	-	-	-	-
Quartile 3 (200-318)	1.21 (0.44, 3.36)	0.71	1.18 (0.42, 3.31)	0.75
Quartile 4 (>318)	0.87 (0.31, 2.44)	0.79	0.88 (0.31, 2.50)	0.81
Leucine				
Quartile 1 (<171)	0.60 (0.22, 1.64)	0.32	0.55 (0.20, 1.53)	0.25
Quartile 2 (171-222)^	-	-	-	-
Quartile 3 (227-465)	0.35 (0.12, 1.00)	0.05	0.32 (0.11, 0.93)	0.04
Quartile 4 (>465)	0.53 (0.18, 1.51)	0.23	0.56 (0.19, 1.64)	0.29
Phenylalanine				
Quartile 1 (<61)	1.09 (0.38, 3.15)	0.87	1.08 (0.37, 3.16)	0.89
Quartile 2 (61-73)^	-	-	-	-
Quartile 3 (74-109)	1.20 (0.43, 3.37)	0.73	1.13 (0.40, 3.22)	0.82
Quartile 4 (>109)	1 (0.34, 2.92)	1.00	1.12 (0.37, 3.35)	0.84
Tyrosine				
Quartile 1 (<24)	2.24 (0.80, 6.28)	0.13	2.03 (0.70, 5.90)	0.20
Quartile 2 (24-47)	1.74 (0.60, 5.03)	0.31	1.68 (0.57, 4.93)	0.35
Quartile 3 (49-86)^	-	-	-	-
Quartile 4 (>86)	1.16 (0.39, 3.43)	0.79	1.18 (0.39, 3.52)	0.77
Methionine				
Quartile 1 (<20)	0.77 (0.28, 2.10)	0.61	0.80 (0.29, 2.19)	0.66
Quartile 2 (20-30)	1.38 (0.50, 3.87)	0.54	1.46 (0.52, 4.15)	0.47
Quartile 3 (31-67)^	-	-	-	-
Quartile 4 (>67)	0.71 (0.25, 2.02)	0.53	0.78 (0.27, 2.26)	0.65
Citrulline				
Quartile 1 (<12)	0.75 (0.27, 2.05)	0.58	0.66 (0.23, 1.85)	0.43
Quartile 2 (12-16)^	-	-	-	-
Quartile 3 (17-22)	0.58 (0.21, 1.58)	0.28	0.54 (0.20, 1.50)	0.24
Quartile 4 (>22)	0.52 (0.19, 1.41)	0.20	0.51 (0.19, 1.41)	0.19

OR, Odds ratio; CI, Confidence interval.

*Adjusted for sex, hospital site and SGA. ^Denotes reference group.

Supplementary Table S8. Odds ratios of clinical outcome by amino acid brand in the placebo group.

**Clinical outcome**	**Amino acid brand**	**OR (95% CI)**	***p*-value**
Intraventricular haemorrhage			
Primène	38/116 (32.8%)	0.85 (0.47-1.54)	0.60
Trophamine	27/92 (29.4%)		
Adjusted for hospital		0.58 (0.15-2.26)	0.43
Bronchopulmonary dysplasia			
Primène	65/99 (65.7%)	1.52 (0.80-2.90)	0.20
Trophamine	61/82 (74.4%)		
Adjusted for hospital		0.71 (0.18-2.87)	0.64
Retinopathy of prematurity			
Primène	17/104 (16.4%)	0.95 (0.43-2.09)	0.90
Trophamine	13/83 (15.7%)		
Adjusted for hospital		1.06 (0.25-4.41)	0.94
Necrotising enterocolitis			
Primène	16/116 (13.8%)	0.76 (0.33-1.77)	0.53
Trophamine	10/92 (10.9%)		
Adjusted for hospital		2.37 (0.20-28.67)	0.50
Patent ductus arteriosus			
Primène	48/114 (42.1%)	0.95 (0.54-1.67)	0.86
Trophamine	36/88 (40.9%)		
Adjusted for hospital		4.25 (0.43-42.19)	0.22
Early onset sepsis – culture proven			
Primène	5/116 (4.3%)	0.25 (0.03-2.15)	0.17
Trophamine	1/91 (1.1%)		
Adjusted for hospital		2.36e-7	0.99
Early onset sepsis – probable			
Primène	14/116 (12.1%)	4.99 (2.48-10.03)	<0.0001
Trophamine	37/91 (40.7%)		
Adjusted for hospital		2.36e-7	0.99
Late onset sepsis – culture proven			
Primène	31/116 (26.7%)	1.56 (0.86-2.82)	0.14
Trophamine	33/91 (36.3%)		
Adjusted for hospital		2.55 (0.60-10.84)	0.21
Late onset sepsis – probable			
Primène	32/116 (27.6%)	3.06 (1.72-5.47)	0.0001
Trophamine	49/91 (53.9%)		
Adjusted for hospital		1.80 (0.26-12.23)	0.55
Death before discharge			
Primène	24/113 (21.2%)	0.66 (0.32-1.36)	0.25
Trophamine	14/93 (15.1%)		
Adjusted for hospital		2.50 (0.21-30.12)	0.47

Data are presented as number (pecent) and odds ratio (95% CI). Significant p-value <0.01 shown in red.

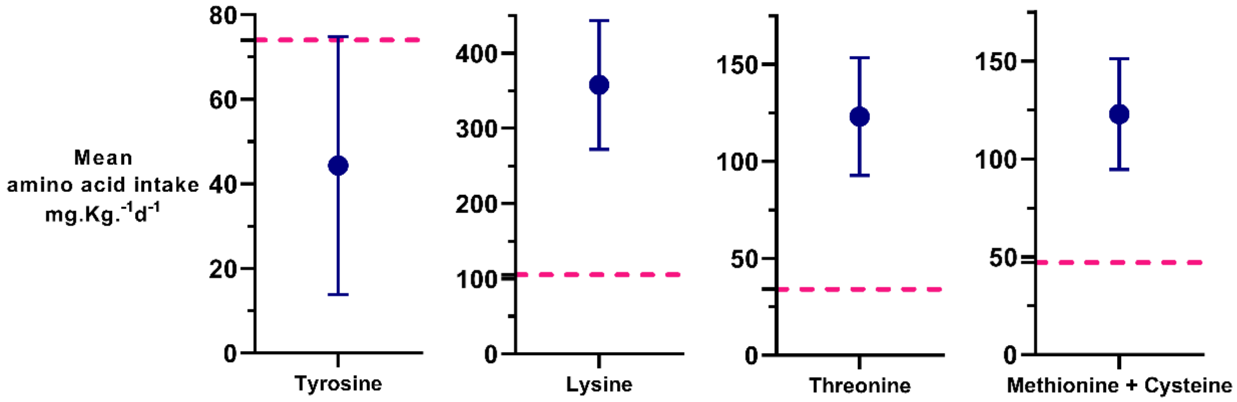

Supplementary Figure S1

Week 1 mean amino acid intakes in the ProVIDe cohort compared with ESPGHAN 2018 recommended intakes. Data are mean (SD). Pink dotted horizontal line denotes the recommended intake: Tyrosine 74 mg.Kg^{-1}.day^{-1} [8]; Lysine 105 mg.Kg^{-1}.day^{-1} [9]; Threonine 38 mg.Kg^{-1}.day^{-1} [10]; Methionine + cysteine 47 mg.Kg^{-1}.day^{-1} [11]

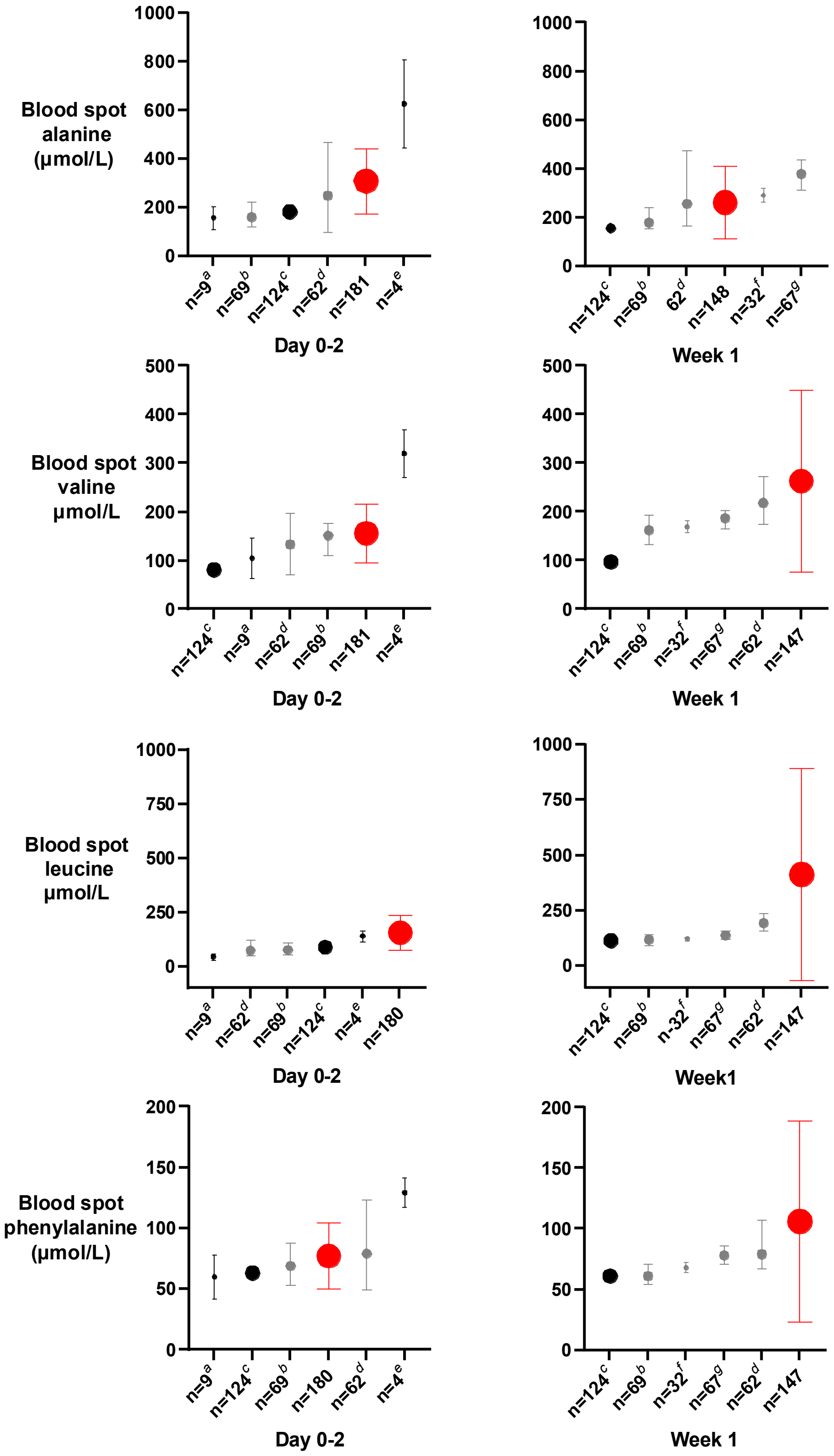
Blood spot alanine (µmol/L)
1000
800
600
400
200
0
n=9a
n=69b
n=124c
n=62d
n=181
n=4e
Day 0-2
1000
800
600
400
200
0
n=124c
n=69b
62d
n=148
n=32f
n=67g
Week 1
Blood spot valine µmol/L
500
400
300
200
100
0
n=124c
n=9a
n=62d
n=69b
n=181
n=4e
Day 0-2
500
400
300
200
100
0
n=124c
n=69b
n=32f
n=67g
n=62d
n=147
Week 1
Blood spot leucine µmol/L
1000
750
500
250
0
n=9a
n=62d
n=69b
n=124c
n=4e
n=180
Day 0-2
1000
750
500
250
0
n=124c
n=69b
n-32f
n=67g
n=62d
n=147
Week1
Blood spot phenylalanine (µmol/L)
200
150
100
50
0
n=9a
n=124c
n=69b
n=180
n=62d
n=4e
Day 0-2
200
150
100
50
0
n=124c
n=69b
n=32f
n=67g
n=62d
n=147
Week 1

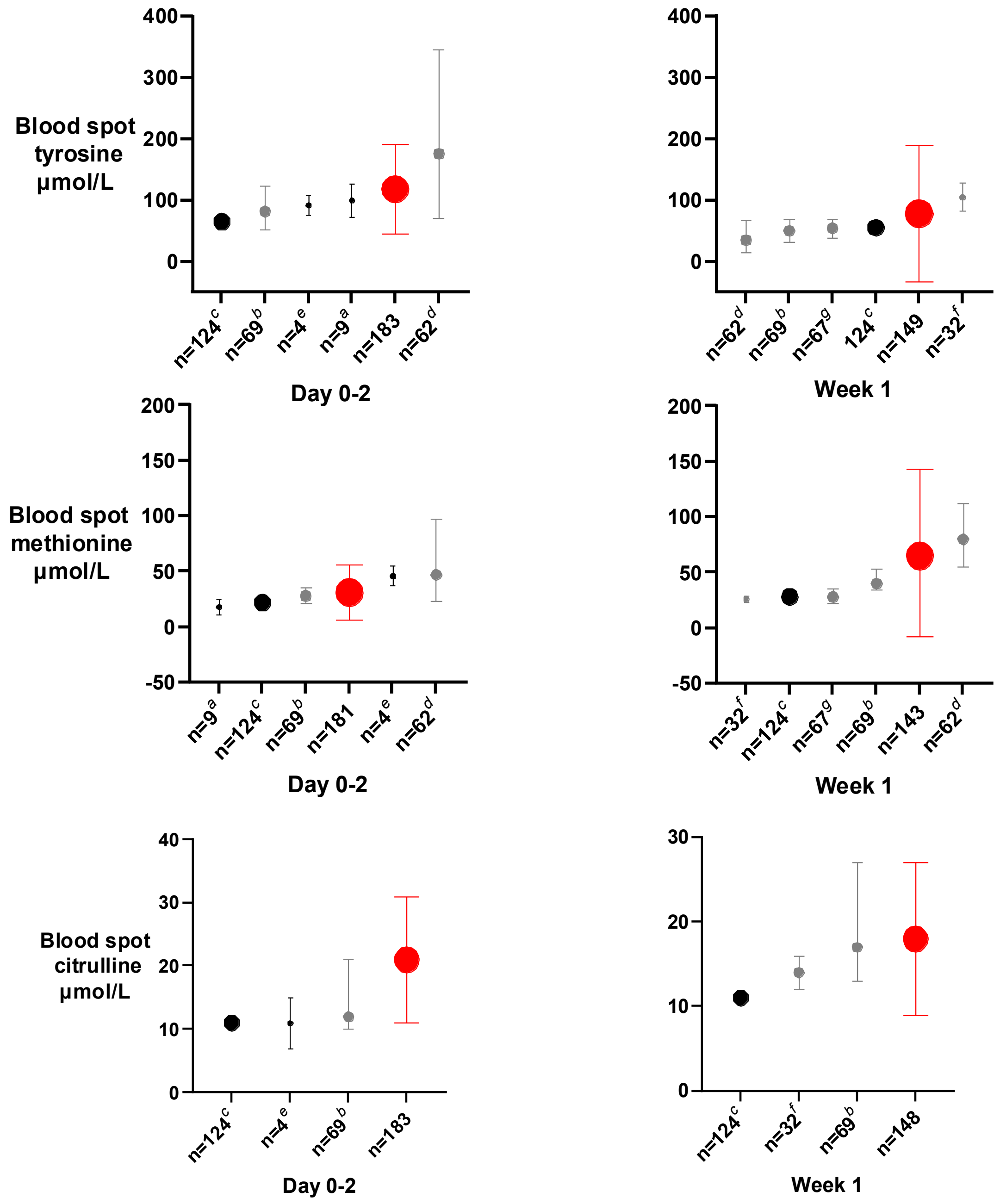

Supplementary Figure S2 Blood spot/plasma amino acid concentrations on day 0-2 and week 1 in the ProVIDe trial and other preterm studies.

Size of circle represents sample size. Red circle represents the ProVIDe trial mean (SD). Black circle represents mean and grey circle represents median. [a][12] mean (SD). [b][13]median (IQR). [c][14] mean. [d][15] median (10th - 90th percentile). [e][16] mean (SD). [f][17] median (SD). [g][18] median (IQR).

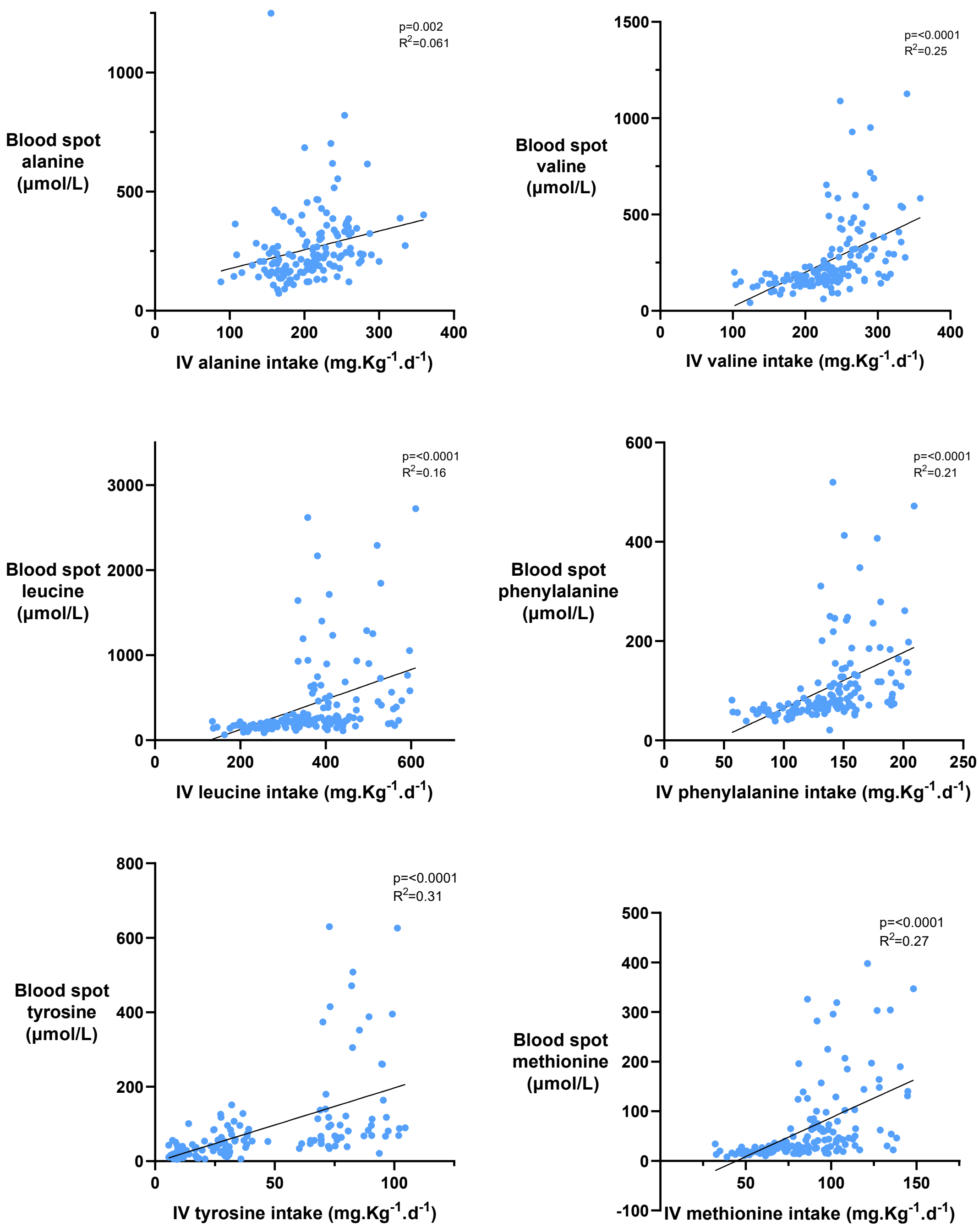

Supplementary Figure S3. Relationship between amino acid intake in week 1 and Week 1 blood spot amino acid concentrations.

Supplementary References

1. Papile, L.A.; Burstein, J.; Burstein, R.; Koffler, H. Incidence and evolution of subependymal and intraventricular hemorrhage: a study of infants with birth weights less than 1,500 gm. *J. Pediatr.* **1978**, *92*, 529-534.
2. Pajak, A.; Krolak-Olejnik, B.; Szafranska, A. Early hypophosphatemia in very low birth weight preterm infants. *Adv Clin Exp Med* **2018**, 10.17219/acem/70081, doi:10.17219/acem/70081.
3. Bell, M.J.; Ternberg, J.L.; Feigin, R.D.; Keating, J.P.; Marshall, R.; Barton, L.; Brotherton, T. Neonatal necrotizing enterocolitis. Therapeutic decisions based upon clinical staging. *Ann. Surg.* **1978**, *187*, 1-7.
4. Bayley, N. *Bayley Scales of Infant and Toddler Development Administration Manual*, 3 ed.; TX: Harcourt Assessment: San Antonio, 2006.
5. Ansell, J.M.; Wouldes, T.A.; Harding, J.E.; group, C.S. Executive function assessment in New Zealand 2-year olds born at risk of neonatal hypoglycemia. *PLoS One* **2017**, *12*, e0188158, doi:10.1371/journal.pone.0188158.
6. Baron, I.S. Test review: Behavior rating inventory of executive function. *Child Neuropsychol.* **2000**, *6*, 235-238, doi:10.1076/chin.6.3.235.3152.
7. Doyle, L.W. Changing availability of neonatal intensive care for extremely low birthweight infants in Victoria over two decades. *Med. J. Aust.* **2004**, *181*, 136-139, doi:doy10817_fm [pii].
8. Roberts, S.A.; Ball, R.O.; Moore, A.M.; Filler, R.M.; Pencharz, P.B. The effect of graded intake of glycyl-L-tyrosine on phenylalanine and tyrosine metabolism in parenterally fed neonates with an estimation of tyrosine requirement. *Pediatr. Res.* **2001**, *49*, 111-119, doi:10.1203/00006450-200101000-00022.
9. Chapman, K.P.; Courtney-Martin, G.; Moore, A.M.; Langer, J.C.; Tomlinson, C.; Ball, R.O.; Pencharz, P.B. Lysine requirement in parenterally fed postsurgical human neonates. *Am. J. Clin. Nutr.* **2010**, *91*, 958-965, doi:10.3945/ajcn.2009.28729.
10. Chapman, K.P.; Courtney-Martin, G.; Moore, A.M.; Ball, R.O.; Pencharz, P.B. Threonine requirement of parenterally fed postsurgical human neonates. *Am. J. Clin. Nutr.* **2009**, *89*, 134-141, doi:10.3945/ajcn.2008.26654.
11. Courtney-Martin, G.; Chapman, K.P.; Moore, A.M.; Kim, J.H.; Ball, R.O.; Pencharz, P.B. Total sulfur amino acid requirement and metabolism in parenterally fed postsurgical human neonates. *Am. J. Clin. Nutr.* **2008**, *88*, 115-124, doi:10.1093/ajcn/88.1.115.
12. Van Goudoever, J.B.; Colen, T.; Wattimena, J.L.; Huijmans, J.G.; Carnielli, V.P.; Sauer, P.J. Immediate commencement of amino acid supplementation in preterm infants: effect on serum amino acid concentrations and protein kinetics on the first day of life. *J Pediatr* **1995**, *127*, 458-465, doi:10.1016/s0022-3476(95)70083-8.
13. Poindexter, B.B.; Ehrenkranz, R.A.; Stoll, B.J.; Koch, M.A.; Wright, L.L.; Oh, W.; Papile, L.A.; Bauer, C.R.; Carlo, W.A.; Donovan, E.F., et al. Effect of parenteral glutamine supplementation on plasma amino acid concentrations in extremely low-birth-weight infants. *Am. J. Clin. Nutr.* **2003**, *77*, 737-743.
14. Liu, D.; Wang, L.; Shen, H.; Han, L.; Wang, Y.; He, Z. Dynamic changes in blood amino acid concentrations in preterm infants in different nutritional periods. *Asia Pac. J. Clin. Nutr.* **2020**, *29*, 803-812, doi:10.6133/apjcn.202012_29(4).0016.
15. Blanco, C.L.; Gong, A.K.; Green, B.K.; Falck, A.; Schoolfield, J.; Liechty, E.A. Early changes in plasma amino acid concentrations during aggressive nutritional therapy in extremely low birth weight infants. *J. Pediatr.* **2011**, *158*, 543-548 e541, doi:10.1016/j.jpeds.2010.09.082.
16. Pittard, W.B., 3rd; Geddes, K.M.; Picone, T.A. Cord blood amino acid concentrations from neonates of 23-41 weeks gestational age. *JPEN J. Parenter. Enteral Nutr.* **1988**, *12*, 167-169, doi:10.1177/0148607188012002167.
17. Becker, R.M.; Wu, G.; Galanko, J.A.; Chen, W.; Maynor, A.R.; Bose, C.L.; Rhoads, J.M. Reduced serum amino acid concentrations in infants with necrotizing enterocolitis. *J. Pediatr.* **2000**, *137*, 785-793, doi:10.1067/mpd.2000.109145.
18. Morgan, C.; Burgess, L. High protein intake does not prevent low plasma levels of conditionally essential amino acids in very preterm infants receiving parenteral nutrition. *J Parenter. Enteral Nutr.* **2017**, *41*, 455-462, doi:10.1177/0148607115594009.
